# Integrative and Conjugative Elements of *Helicobacter pylori* Are Hypothetical Virulence Factors Associated With Gastric Cancer

**DOI:** 10.3389/fcimb.2020.525335

**Published:** 2020-10-19

**Authors:** Eduardo Mucito-Varela, Gonzalo Castillo-Rojas, Juan J. Calva, Yolanda López-Vidal

**Affiliations:** ^1^Departamento de Microbiología y Parasitología, Programa de Inmunología Molecular Microbiana, Facultad de Medicina, Universidad Nacional Autónoma de México (UNAM), Mexico City, Mexico; ^2^Department of Infectious Diseases, Instituto Nacional de Ciencias Médicas y Nutrición “Salvador Zubirán” (INCMNSZ), Mexico City, Mexico

**Keywords:** *Helicobacter pylori*, gastric cancer, virulence factor, integrative and conjugative elements (ICE), comparative genomics

## Abstract

*Helicobacter pylori* is a bacteria with high genome plasticity that has been associated with diverse gastric pathologies. The genetic diversity of this bacteria has limited the characterization of virulence factors associated with gastric cancer (GC). To identify potentially helpful disease biomarkers, we compared 38 complete genomes and 108 draft genomes of *H. pylori* isolated worldwide from patients with diverse gastric pathologies and 53 draft genomes of *H. pylori* isolated from Mexican patients with GC, intestinal metaplasia, gastritis, peptic ulcer, and dyspepsia. *H. pylori* strains isolated from GC were 3–11 times more likely to harbor any of seven genes encoded within an integrative and conjugative element (ICE) than *H. pylori* isolated from subjects with other gastric pathologies. We tested the cytopathic effects on AGS cells of selected *H. pylori* strains with known cytotoxin-associated gene pathogenicity island (*cag*-PAI) and ICE status (*H. pylori* strains 29CaP, 29CaCe, 62A9, 7C, 8822, and 26695) and the histopathological damage of *H. pylori* 29CaP and 62A9 in a mouse model. *H. pylori* 29CaP, which harbors a complete ICEHptfs3 but lacks *cag*-PAI, elicited distinctive morphology changes and higher histopathological scores compared with other *H. pylori* strains carrying *cag*-PAI and hybrid ICE with incomplete TFSS. The presence of intact segments of ICE regions might be a risk factor to develop GC that needs to be addressed in future studies.

## Introduction

*Helicobacter pylori* is a gram-negative bacterium that inhabits the gastric mucosa of nearly 50% of the world's population (Polk and Peek, 2010). Infection by *H. pylori* produces chronic gastritis and increases the risk of peptic ulcer, gastric cancer, and mucosa-associated lymphoid tissue (MALT) lymphoma. Gastric cancer is a major public health concern, as it represents the sixth leading cause of death worldwide (Ferlay et al., 2015).

Gastric pathogenesis is caused by the interaction between *H. pylori* virulence factors and gastric epithelial cells. These interactions inhibit the immune response and alter inflammatory and carcinogenic pathways (Dorer et al., 2009; Polk and Peek, 2010). The presence of the vacuolating cytotoxin A (VacA) and the cytotoxin-associated gene pathogenicity island (*cag*-PAI) with its CagA effector protein, as well as their polymorphic variation, explains many mechanisms leading to gastric cancer, mainly by disrupting cell signaling involved in cell adhesion, cell proliferation, and cell differentiation (Rivas-Ortiz et al., 2017). However, the presence of these two genes is not enough to predict the outcome of *H. pylori* infection.

Studies comparing the characteristics of the *H. pylori* genomes isolated from different human populations show differences in size, gene number, and G+C content. Additionally, several segments are acquired by recombination and cause divergence of some genes in certain *H. pylori* phylogroups (Kawai et al., 2011; Yahara et al., 2013; Dong et al., 2014). These observations raise the question of whether there could be specific interactions between a determined phylogroup of *H. pylori* and human hosts in a given human population. Some studies have reported differences in gene content between the genomes of *H. pylori* isolated from gastric cancer, when compared with the genomes of *H. pylori* isolated from different gastric pathologies, mainly in genes encoded in genomic islands, such as restriction–modification systems, transposases, and genes from type IV secretion system (T4SS) (McClain et al., 2009; Romo-González et al., 2009; You et al., 2012). The genomic islands of *H. pylori* were previously known as plasticity zones because of its variable gene content among strains; however, they are currently well-characterized as integrative and conjugative elements (ICEs) due to the capability of conjugative transfer and recombination with the genome (Fischer et al., 2014). These ICEs are formally named ICEHptfs3 and ICEHptfs4, indicating the presence of a T4SS different from the *cag*-PAI and the competence system (Fischer et al., 2014). Each ICE island encodes between 35 and 38 genes, 13 of which are the only genes conserved among all ICEs. Those conserved genes are members of a T4SS (*VirB, D*, and *C* genes) and other genes involved in element replication and mobilization like XerT integrase and a topoisomerase (TopA) (Delahay et al., 2018). Therefore, the sizes of ICEs are variable, although ICEHptfs3 islands have a length of about 37.6 or 46 kb, depending on the presence of pz21-23 orthologs, whereas complete ICEHptfs4a, ICEHptfs4b, and ICEHptfs4c usually comprise about 40.7, 39.4, and 39.3 kb, respectively. These subtypes are defined according to the presence of polymorphic genes hpp12_446/hpg27_981 and hpp12_444-445/hpg27_982, described first in the ICEs of *H. pylori* P12 and G27 (Fischer et al., 2014). A recent study using *H. pylori* genomes of strains isolated worldwide showed that ICEHptfs3 is commonly found as fragments and is rarely complete, unlike ICEHptfs4. In addition, ICEHptfs3 is more frequent in *H. pylori* with the HpAfrica1 population structure, which means the presence of this element correlates with a greater African genetic ancestry (Delahay et al., 2018).

The evaluation of ICE functionality has been measured by its ability to induce inflammatory response upon contact with human gastric adenocarcinoma-derived (AGS) cells using *H. pylori* strains harboring either wild type or mutant ICE elements (Kersulyte et al., 2009; Silva et al., 2017). In another experimental approach, the functionality of ICE was determined by its capability to transfer selected genetic markers between *H. pylori* strains (Fischer et al., 2010). These reports suggest that the functionality of ICE in *H. pylori* might depend on the completeness of its T4SS.

In this study, we looked for genes potentially associated with gastric cancer based on *in silico* analysis looking for genes present at high frequencies in *H. pylori* isolated from this pathology. This approach was done by comparing a set of *H. pylori* genomes of strains isolated from different gastric pathologies and different population structures. We found a correlation between the genes associated with gastric cancer and the presence of ICEs, leading to hypothesize that the presence of these genomic regions might encode virulence factors involved in the pathogenesis of cancer. The infection experiments carried in cell culture and mice gave insights into the effects caused by *H. pylori* strains carriers of ICEHptfs3 that must be further characterized in future studies.

## Materials and Methods

### *H. pylori* Genome Sequences

The sequences of *H. pylori* genomes and plasmids used in this study were selected from the National Center for Biotechnology Information Genome database, based on the availability of clinical information regarding their associated gastric pathology in the literature or genome descriptions (Table 1 and Supplementary Table 1). The genomes were divided into three datasets: (1) Exploratory data set, comprising the first three *H. pylori* genome sequences of strains isolated from Mexican patients; (2) Validation set 1, including 38 *H. pylori* complete genome sequences from strains isolated worldwide and two complete *H. pylori* genomes isolated in Mexico; and (3) Validation set 2, consisting of 139 genome sequences from strains isolated worldwide, including three *H. pylori* sequences from Mexico and several other draft sequences. Additionally, 34 draft genomes of *H. pylori* isolated in Mexico (Thorell et al., 2017) were analyzed to further validate the observations only in strain isolates in Mexico. The population structure of the genomes was obtained after reviewing the literature. When the information was not available, it was inferred by average nucleotide identity (ANI)-BLAST clustering.

**Table 1:** *H. pylori* genomes used for comparative analysis in the detection of genes associated with gastric cancer from exploratory and validation 1 genome sets.

***H. pylori* strain**	**Accession number**	**Pathology**	***cag*-PAI (EPIYA)**	**VacA**	**ICE Type**	**MLST group**
29CaP (a)	NZ_CP012907.1	GC	–	s2/i2/m2	ICEHptfs3, ICEHptfs4 (P)	hpEurope
CG-IMSS-2012 (a, b)	NZ_AWUL00000000.1	GC	+ (ABC)	s1b/i1/m1	ICEHptfs3 (*)	hpEurope
PeCan4	NC_014555.1, NC_014556.1	GC*	+ (ABBC)	s1a/i1/m1	ICEHptfs4	hpEurope
ELS37	NC_017063.1, NC_017064.1	GC*	+ (ABC)	s1b/i1/m1	ICEHptfs3 (*)	hpEurope
UM037	NC_021217.3	GC	+ (ABC)	NA	ICEHptfs3 (*)	hpEurope
XZ274	NC_017926.1, NC_017919.1	GC	+ (ABBD)	s1a/i1/m1	ICEHptfs4 (P)	hspEAsia
F32	NC_017366.1, NC_017370.1	GC	+ (ABD)	s1a/i1/m1	ICEHptfs3 (P*)	hspEAsia
F57	NC_017367.1	GC	+ (ABD)	s1a/i1/m1	ICEHptfs4	hspEAsia
OK310	NC_020509.1, NC_020556.1	GC	+ (ABCC)	s1a/i1/m1	ICEHptfs3 (*)	hspEAsia
PeCan18	NC_017742.1	GC*	+ (ABC)	s1b/i1/m1	ICEHptf3	hpAfrica1
HPAG1	NC_008086.1, NC_008087.1	CAG	+ (ABBC)	s1b/i1/m1	–	hpEurope
oki102	NZ_CP006820.1	CAG	+ (ABC)	s1a/i1-i2/m2	ICEHptfs4	NR
oki112	NZ_CP006821.1	CAG	+ (ABC)	s1a/i1-i2/m2	ICEHptfs4+	NR
oki128	NZ_CP006822.1	CAG	–	s2/i2/m2	–	NR
oki422	NZ_CP006824.1	CAG	+ (ABC)	s1a/i1-i2/m2	ICEHptfs4	NR
P12	NC_011498.1, NC_011499.1	DU	+ (ABCC)	s1a/i1/m1	ICEHptfs3, ICEHptfs4	hpEurope
F30	NC_017365.1, NC_017369.1	DU	+ (ABD)	s1a/i1/m1	–**	hspEAsia
OK113	NC_020508.1	DU	+ (ABD)	NA	ICEHptfs3 (*)	hspEAsia
UM032	NC_021215.3	PU	+ (ABD)	s1a/i1/m2	–**	hspEAsia
UM066	NC_021218.3	PU	+ (ABD)	s1a/i1/m2	Hybrid ICEHptfs3/ICEHptfs4	hspEAsia
Shi470	NC_010698.2	GU*	+ (ABC/CC)	s1b/i1/m1	ICEHptfs4	hspAmerind
J99	NC_000921.1	DU	+ (BC)	s1b/i1/m1	Hybrid ICEHptfs3/ICEHptfs4	hpAfrica1
908	NC_017357.1	DU	+ (ABC)	NA	ICEHptfs4 (P)	hpAfrica1
oki154	NZ_CP006823.1	DU	–	s2/i2/m2	ICEHptfs3 (*)	NR
oki673	NZ_CP006825.1	GU	–	s2/i2/m2	ICEHptfs3 (*)	NR
oki828	NZ_CP006826.1	DU	–	NA	ICEHptfs3 (*)	NR
oki898	NZ_CP006827.1	DU	+ (ABC)	s1a/i1-i2/m2	ICEHptfs4	NR
7C (a)	NZ_CP012905.1, NZ_CP012906.1	G	–	s2/i2/m2	Hybrid ICEHptfs3/ICEHptfs4	hpEurope
26695	NC_000915.1	G	+ (ABC)	s1a/i1/m1	Hybrid ICEHptfs3/ICEHptfs4 (P)	hpEurope
SJM180	NC_014560.1	G*	+ (ABC)	s1b/i1/m1	Hybrid ICEHptfs3/ICEHptfs4	hpEurope
F16	NC_017368.1	G	+ (ABD)	s1a/i1/m1	–	hspEAsia
v225d	NC_017355.1, NC_017383.1	G	+ (ABCC)	s1a/i1/m1	–	hspAmerind
Puno120	NC_017378.1, NC_017377.1	G*	+ (ABCC)	s1b/i1/m1	Hybrid ICEHptfs3/ICEHptfs4 (P)	hspAmerind
Puno135	NC_017379.1	G*	+ (ABCC)	s1b/i1/m1	Hybrid ICEHptfs3/ICEHptfs4 (P)	hspAmerind
Santal49	NC_017376.1, NC_017380.1	NP*	+ (ABC)	s1a/i1/m2	ICEHptfs4	hpAsia2
B38	NC_012973.1	MALT	–	s2/i1-i2/m2	–	hpEurope
Hp238	NZ_CP010013.1	MALT	+ (ABD)	NA		NR
ML1	NZ_AP014710.1	MALT	+ (ABD)	s1a/i1/m2	ICEHptfs3 (*)+	NR
ML3	NZ_AP014712.1, NZ_AP014713.1	MALT	+ (ABD)	s1a/i1/m1	ICEHptfs3 (*), ICEHptfs3 (P)	NR

*a, Genomes included in an exploratory dataset*.

*b, Genomes not included in validation set 1*.

*GC, Gastric cancer; G, Gastritis; DU, Duodenal ulcer; CAG, Chronic atrophic gastritis; MALT, Mucosa associated lymphoid tissue lymphoma; GA, Gastric atrophy; GU, Gastric ulcer; PU, Peptic ulcer; NP, Asymptomatic*.

*NA, No sequence available due to gene disruption*.

*NR, Not reported*.

*(P), partial sequence*.

(*)Evidence of recombination with ICEHptfs4 in some regions.

**A small segment of ICEHptfs4 first quarter was present.

### *H. pylori* Genome Sequencing

Eighteen *H. pylori* genomes isolated from Mexican patients with different gastric pathologies were sequenced to further validate previous results (Supplementary Table 2). All the *H. pylori* strains were cultured in Casman agar (BD BIOXON) plates supplemented with 10% horse serum (American Type Culture Collection 30-2040). The plates were incubated at 37°C for 48 h in microaerophilic conditions. The genomic DNA of the *H. pylori* strains was obtained with the cetyltrimethylammonium bromide–phenol–chloroform method (Wilson, 1997). The genomic DNA of each strain was sequenced by synthesis using Illumina Miseq. Libraries of 500 and 150 bp were prepared and sequenced in parallel to a depth of 100×, approximately. The readings obtained were analyzed with FastQC (Andrews, 2010) and SolexaQA (Cox et al., 2010) to verify their quality. Velvet (Zerbino and Birney, 2008) was used to *de novo* assemble the readings and create a set of scaffolds using k-mer sizes 31–63. The best assemblies obtained from each genome were aligned to one another using NUCmer (Kurtz et al., 2004) to obtain the longest contigs possible and to fill in the gaps in the scaffolds. The final assemblies were checked for continuous coverage and sequencing depth at 10× after aligning the entire reads with Bowtie (Langmead et al., 2009). The genomes were annotated automatically using MG-RAST (Glass and Meyer, 2011) for comparative analysis.

### Identification of Protein Orthologous Groups

The selected datasets of genomes were analyzed independently using GET_HOMOLOGUES v1.2 (Contreras-Moreira and Vinuesa, 2013) to determine protein orthologous groups (POGs). The default parameters to look for sequence similarities in blastp were chosen. To cluster the orthologous proteins, the OrthoMCL and COGtriangles algorithms were used with the default parameters and no size restriction (option –t 0).

### Identification of Common Proteins in Gastric Cancer-Associated *H. pylori* Strains

The clusters of orthologous proteins were analyzed with the script *compare_clusters.pl* to obtain pan-genome matrices. The pan-genome matrices were interrogated using in-house made R scripts to obtain lists of POGs specific to each genome or shared between two or more. We interrogated the proteins present in at least 60% of the *H. pylori* genomes from gastric cancer isolates and absent in the same proportion from the genomes of other gastric pathologies. To test whether the frequency was higher in the gastric cancer genomes than in the non-cancer ones, a chi-square test with continuity correction was used with the prop.test function of the R stats package v3.4.0 (R Core Team, 2016). We also identified gastric cancer-related POGs obtained from all, two, or single genome datasets.

### *H. pylori* Integrative and Conjugative Element Analysis

The entire region of four types of ICEs was selected according to Fischer et al. (2014). The ICE sequences were aligned with the *H. pylori* genomes using PROmer v3.07 included in MUMmer 3.23 (Kurtz et al., 2004) to look for similar genomic regions. The coordinates of the alignments were used to obtain the sequence of the regions. Then, a multiple sequence alignment was performed using Mauve version 2.4.0 with the default parameters, and the structure of ICEs in complete genomes was analyzed and compared. To visualize the length and position of the alignments in draft genomes, the alignment results were plotted using mummerplot v3.5 (MUMmer 3.23) (Kurtz et al., 2004) with each genome as a reference. The alignment results were scored as “potentially complete ICE” and “incomplete ICE” for genomes with ICE coverage greater or <50%, respectively. This cutoff was used to increment the probability to find an ICE that had few recombination events that could lead to deleterious effects or loose.

Once the coordinates of the ICE regions in the *H. pylori* genomes isolated in Mexico were located, we identified the homologs of the T4SS using a Blast protein approach. Then, tables or graphics were generated to visualize the results.

### Genome Clustering of *H. pylori* Isolates Worldwide by Average Nucleotide Identity

Genomes were clustered using whole-genome ANI to find relationships between genomes. After obtaining the FASTA sequences, the genomes were clustered by ANI using the BLAST (ANI-BLAST) and NUCmer options of the calculate_any.py script developed by Leighton Pritchard at The James Hutton Institute (available at https://github.com/widdowquinn/scripts/blob/master/bioinformatics/calculate_ani.py). In brief, pairwise comparisons were performed to get sequence fragments that covered at least 70% of the query sequence and that had at least 30% nucleotide identity along the full query sequence. The average value of the percent identity of these fragments was arranged in a matrix that was graphed with the “gplots” package in R.

### Evaluation of Cytopathic Effect on AGS Cells

AGS cells were cultured in Roswell Park Memorial Institute (RPMI) medium 1640 (Gibco, Life Technologies) with 10% fetal bovine serum (FBS, Gibco, Life technologies) in a humidified 5% carbon dioxide atmosphere at 37°C. After AGS growth reached 100% confluence, the cells were treated for 3 min with 0.25% trypsin-ethylenediaminetetraacetic acid (Gibco, Life Technologies) at 37°C. The cells were concentrated by centrifugation at 4°C, then washed with 1× PBS and resuspended with RPMI-FBS medium. Immediately, the AGS cells were spiked on 24-well plates at a concentration of 1.2 × 10^5^ cell/well and incubated for 24 h.

For the infection, *H. pylori* strains (29CaP, 8822, 7C, 62A9, and 26695) were grown for 24 h in Brucella broth (BBL Brucella Broth, BD) supplemented with 0.4% cyclodextrin-B (Sigma-Aldrich) at 37°C in agitation at 150 rpm (Orbital Shaker, LAB-LINE). The bacteria were concentrated by centrifugation (SORVALL SUPER T 21) at 4,000 rpm for 5 min at 4°C, and they were washed once with RPMI-FBS medium. We prepared bacterial suspensions containing 1.5 × 10^8^ colony-forming unit (CFU)/ml in RPMI-FBS medium, and then we inoculated three wells per strain with 12 × 10^6^ bacterial cells to reach a multiplicity of infection of 1:100 (100 bacteria per 1 AGS cell). Quality control *H. pylori* was performed by catalase, oxidase, and Gram staining before they were used as inoculum; the predominant bacillary morphology of *H. pylori* was required because this is the naturally infective and viable form.

After 48-h postinfection, the morphology of the AGS cells was observed using an inverted microscope (Leica DMIL). The cell morphology of infected cells was compared with the morphology of uninfected cells that consisted of pyramidal-shaped adherent cells, with more than 80% of confluency. Any loss of adherence, cell elongation, or vacuolization was considered as a cytopathic effect. Thereafter, the supernatants and cells were collected and stored at −70°C until further use.

### Evaluation of Histopathological Damage in Mouse Model

All the protocols were approved by the Internal Committee for the Care and Use of Experimental Animals of the Faculty of Veterinary Medicine and Zootechnics, Universidad Nacional Autónoma de México.

*H. pylori* strains 62A9 and 29CaP were cultured on Casman agar plates (BD BIOXON) supplemented with 10% horse serum (American Type Culture Collection 30-2040), under microaerophilic conditions, at a temperature of 37°C. After 4 days of incubation, a bacterial suspension at 1.2 × 10^8^ CFU/ml was created.

BALB/c mice 6–8 weeks old were infected with single doses via the orogastric route, using 1.2 × 10^7^ CFU. For the 29CaP strain, 60 mice were used (40 infected and 20 uninfected). For strain 62A9, 45 mice were used (30 infected and 15 uninfected). For each strain, groups of five to eight mice were killed at days 1, 7, 14, 90, and 180 post-inoculation with 200 mg/kg of Pentobarbital sodium administered intraperitoneally, as indicated in the Official Mexican Norm for guidelines on the reproduction, maintenance, and use of laboratory animals NOM-062-ZOO-1999. The stomachs were collected in buffered formalin at 4% in 1× PBS, embedded in paraffin, and histological sections stained with hematoxylin–eosin were made for histopathological analysis. A trained veterinarian evaluated the histological sections, and histopathological damage was evaluated using the criteria of Rogers (2012).

We monitored all the time of infection, the qualitative presence of *H. pylori* in the stomachs of the infected mice by immunohistochemistry and PCR-hybridization. For immunohistochemistry, a polyclonal mouse anti-*H. pylori* sera (Dako) was used to recognize the bacteria, and a goat anti-rabbit (BIORAD) was used to reveal the immunoreaction with streptavidin (Bio-SB). For PCR-hybridization, the method reported by Castillo-Rojas et al. (2002) was used. Briefly, total DNA was extracted from the stomach tissue, and 16 rRNA gene was amplified, then a second amplification using *H. pylori* specific primers was carried out. The amplicons were transferred to a nylon membrane, and a probe marked with digoxigenin was used to detect the specific amplicons.

### Statistical Analysis

Results of gene frequencies are reported as absolute values and percentages. The rate of diverse *H. pylori* genotypes was compared between *H. pylori* isolates from cancer gastric patients and isolates from subjects with other gastric diseases. The differences between the groups in these percentages were tested with chi-square tests, and the magnitude of association was estimated by calculating the odds ratio value and its 95% confidence interval. Values were considered significant when *p* < 0.05. No adjustment of this value for multiple comparisons was carried out, as this is a hypothesis-generating type of study.

## Results

### Genes Encoded in Integrative and Conjugative Elements Are Present at High Frequency in Gastric Cancer-Associated *H. pylori* Isolates Worldwide

**Figure 1:**
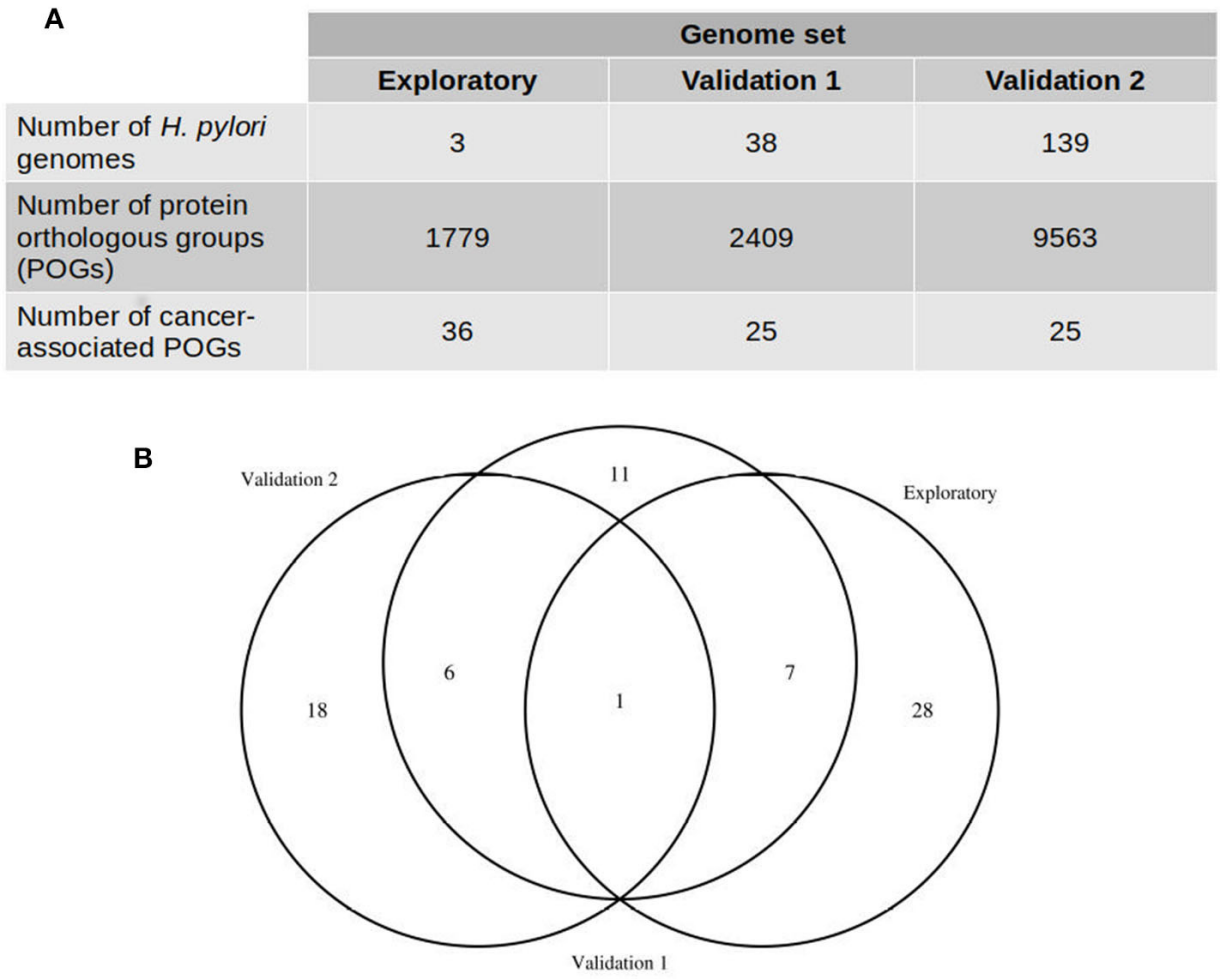
Comparison of protein orthologous groups (POGs) predicted with three *H. pylori* genome datasets. **(A)** Number of genomes included in each genome set and the number of gastric cancer-associated POGs detected. **(B)** Number of POGs detected in the three datasets.

We estimated the pangenome of *H. pylori* using 3, 38, and 139 genome sequences (Table 1 and Supplementary Table 1) by clustering proteins in orthologous groups (POGs) using OrthoMCL and COG algorithms included in GET_HOMOLOGUES v1.2 (Contreras-Moreira and Vinuesa, 2013). We obtained 1,779, 2,409, and 9,563 POGs in the pan-genomes estimated in the three datasets of *H. pylori* genomes, respectively (Figure 1). We considered all gastric cancer-associated POGs (GC-POGs) with a frequency >60% in genomes of *H. pylori* isolated from gastric cancer and those showing less than the same frequency in genomes of *H. pylori* isolated from other gastric pathologies (Table 2). Frequencies between 70 and 100% yielded no results, so we considered 60% as the cutoff. These results were expected due to the plasticity of *H. pylori* genomes; the pangenome analysis showed that at least 30% of the genes were mobile between strains or unique (Uchiyama et al., 2016). Therefore, we selected POGs showing a lower frequency in the other gastric pathologies, increasing the probability of a significant result.

**Table 2:** Association of various genomic profiles of *H. pylori* with gastric cancer.

**GI**	**Function**	**Validation set 1**			**Validation set 2**		
		***H. pylori* isolated from 9 gastric cancer patients**	***H. pylori* isolated from 29 subjects without gastric cancer**	***P-*value**	***H. pylori* isolated from 16 gastric cancer patients**	***H. pylori* isolated from 123 subjects without gastric cancer**	***P-*value**
**Protein also detected in exploratory dataset**							
983282765 (OA23_04715)	Integrase	8 (88.89)	15 (51.72)	0.1091*	14 (87.5)	60 (48.78)	**0.007963**
**Odds ratio (95% confidence interval)**		**7.47 (0.83, 57.51)**			**7.35 (1.6, 33.71)**		
**Proteins detected only in validation sets**							
983282771 (OA23_04830)	DNA topoisomerase I	8 (88.89)	12 (41.38)	**0.03472**	11 (68.75)	73 (59.35)	0.6516
**Odds ratio (95% confidence interval)**		**11.33 (1.25, 102.93)**			**1.51 (0.49. 4.6)**		
983282996 (OA23_06705)	Hypothetical protein	8 (88.89)	12 (41.38)	**0.03472**	12 (75)	72 (58.54)	0.3197
**Odds ratio (95% confidence interval)**		**11.33 (1.25, 102.93)**			**2.13 (0.65, 6.96)**		
447057575 (OA23_04675)	Hypothetical protein	6 (66.67)	15 (51.72)	0.6863	11 (68.75)	26 (21.14)	**0.0001748**
**Odds ratio (95% confidence interval)**		**1.6 (0.33, 7.77)**			**8.21 (2.62, 25.72)**		
447147459	Haloacid dehalogenase	7 (77.78)	13 (44.83)	0.1779	10 (62.5)	71 (57.72)	0.9243
**Odds ratio (95% confidence interval)**		**4.31 (0.76, 24.38)**			**7.32 (0.91, 59.01)**		
983282632	Lipopolysaccharide biosynthesis protein	6 (66.67)	15 (51.72)	0.6863	12 (75)	71 (57.72)	0.2917
**Odds ratio (95% confidence interval)**		**1.87 (0.39, 8.93)**			**2.2 (0.67, 7.2)**		
446412707	Sulfatase	6 (66.67)	10 (34.48)	0.1862	11 (68.75)	51 (41.46)	0.07215
**Odds ratio (95% confidence interval)**		**3.8 (0.78, 18.51)**			**3.11 (1.02, 9.48)**		

The inference of the functions associated with the proteins identified as GC-POG was done by aligning the sequences to the Kyoto Encyclopedia of Genes and Genomes gene databases using BlastKOALA (Kanehisa et al., 2016). This resulted in a high proportion of proteins classified in the functional categories related to nucleotide metabolism and genetic information processing, including restriction–modification systems, DNA replication, and repair proteins (Supplementary Table 3).

We observed that one GC-POG detected in the three datasets of genomes exhibited statistical significance in the dataset conformed by 139 genomes (cancer genomes *n* = 16, non-cancer genomes *n* = 123, *p* < 0.05, chi-square test). This POG also had greater frequency in cancer genomes than gastritis in the dataset conformed by 38 genomes; however, in this dataset, the POG was not statistically significant at the two-sided hypothesis but a greater one-sided hypothesis. The proteins integrating this GC-POG were annotated in some genomes as an integrase. *H. pylori* isolated from gastric cancer patients were 3–11 times more likely to harbor any of seven GC-POGs genes encoded within an ICE than *H. pylori* isolated from subjects with other gastric pathologies (Table 2). Three of the six identified GC-POGs had statistical significance (*p* < 0.05, chi-square test) in either of the datasets; one of these three GC-POGs was formed by proteins annotated as DNA topoisomerase I, and the other two were hypothetical proteins (Table 2).

**Figure 2:**
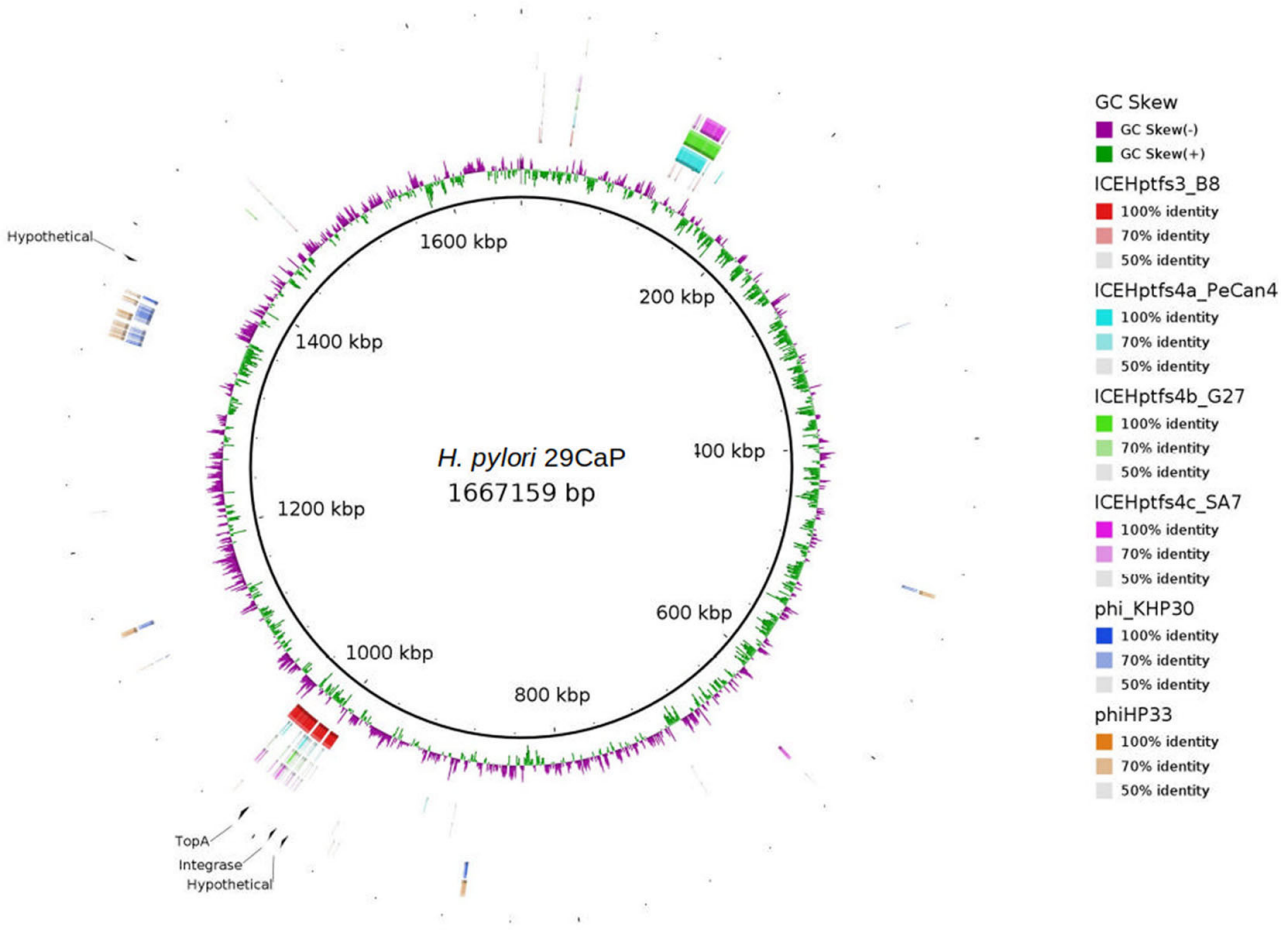
Gastric cancer-associated protein orthologous groups (POGs) mapped on *H. pylori* 29CaP genome. tblastx comparisons of the four ICEs described, and KHP30 and phiHP33 bacteriophage genomes with *H. pylori* 29CaP genome to show the regions where POGs are encoded. Alignments and circular map were obtained with BRIG v0.95.

The integrase and the DNA topoisomerase I were encoded in a region similar to the ICEHptfs3 in the *H. pylori* 29CaP genome, whereas the other two hypothetical proteins were close to this ICE or the prophage region in *H. pylori* 29CaP (Figure 2). The observations allowed us to hypothesize that these mobile elements could play a distinctive role in gastric cancer pathogenesis. To test whether the presence of the ICEs was associated with gastric cancer, we aligned the entire sequences of the four ICE types currently described with the 38 complete genomes included in the validation set 1. We found the presence of ICE element regions in 9/9 of the *H. pylori* genomes from gastric cancer (Supplementary Figure 1A), 3/5 of the *H. pylori* genomes from gastric atrophy (Supplementary Figure 1B), 6/8 of *H. pylori* genomes from gastritis-asymptomatic patients (Supplementary Figure 1C), 9/12 of *H. pylori* genomes from peptic ulcer (Supplementary Figure 1D), and 3/4 *H. pylori* genomes from MALT lymphoma (Supplementary Figure 1E). We observed that ICEHptfs3 was present in 6/9 of the complete genomes of gastric cancer isolates. Also, we could infer the presence of this element in the draft genome of CG-IMSS-2012. The genomes of peptic ulcer and MALToma had 5/12 and 3/4 genomes carrying ICEHptfs3, respectively. ICEHptfs3 was not found in gastritis or chronic atrophic gastritis genomes, which carried instead hybrid ICEs conformed by half of ICEHptfs3 and ICEHptfs4 (Table 1).

### *H. pylori* From Mexican Patients Harbors Different Integrative and Conjugative Element Types

To further validate the previous results, we used a set of 53 draft genomes of *H. pylori* isolated in Mexico from different gastric pathologies, 18 of which were sequenced in this study. We identified regions similar to the four types of ICE using PROmer alignments in all the genomes analyzed. Considering the length and coverage of the alignments, we observed that the genomes of *H. pylori* isolated from gastric cancer, intestinal metaplasia, and peptic ulcer had a greater frequency of potential complete ICEs as compared with the gastritis and dyspepsia *H. pylori* genomes (Table 3). The ICE sequences identified in the *H. pylori* genomes were mostly hybrids between the four types of ICEs, with greater length segments from ICEHptfs3 (Supplementary Table 2). The last third of the ICEHptfs3 was present more frequently in the hybrid ICEs of gastric cancer and premalignant pathologies of genomes of Mexican *H. pylori*.

**Table 3:** Presence of potentially complete ICEs in *H. pylori* draft genomes from strains isolated in Mexico.

**Gastric pathology**	**Total number of *H. pylori* genomes**	**Number of genomes with**	
		**Potentially complete ICE**	**Incomplete ICE**
Gastric cancer	16	9	7
Intestinal metaplasia	10	5	5
Gastritis and chronic gastritis	20	5	15
Peptic ulcer	3	2	1

The integrase and DNA topoisomerase I homologous coding sequences were searched within the orthologous groups determined by the OrthoMCL algorithm in the get_homologues package and tblastx to exclude a miss-annotation. We did not identify any correlation between the presence of these genes and a gastric pathology, as observed in the datasets conformed by the genomes of worldwide *H. pylori* strains.

### Gastric Cancer-Associated *H. pylori* Isolates in Mexico Show an Increase in hpAfrica1 Admixture

**Figure 3:**
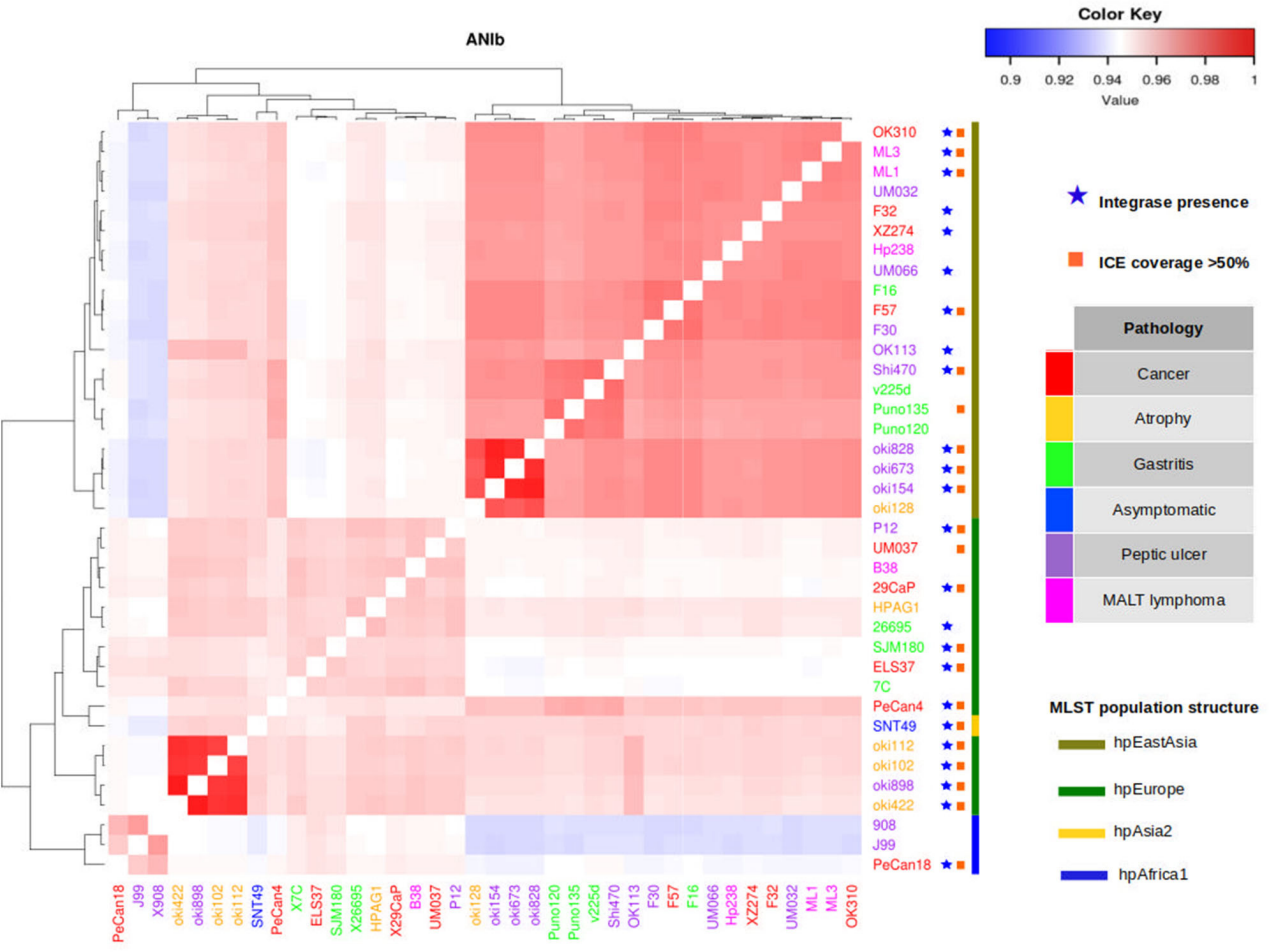
Distribution of integrase and ICE coverage in 38 *H. pylori* complete genomes. Genomes selected were clustered using ANI-BLAST to match their phylogeographic origin. Presence of integrase gene in genomes is marked as a blue star. Genomes with ICE coverage >50% are marked as an orange square. There is no association between phylogeographic origin and presence of elements mentioned, only a high frequency of these elements in genomes of *H. pylori* isolated from gastric cancer.

To study the relationship between the phylogeographic origin and the gastric pathology, we clustered the selected *H. pylori* genomes with ANI-BLAST to infer the phylogeographic origin of the strains not yet reported, including the genomes sequenced in this study (Supplementary Table 1, Supplementary Figure 2). Previously, we had found that the clusters obtained using this method correlated with the phylogeographic origin already reported by MLST typing or fine structure when using the 38 complete genomes in the analysis (Figure 3).

The genomes of *H. pylori* isolated in Mexico clustered in groups ANI-BLAST I, IA, and II, which correlated with the hpEurope and hpAfrica1 MLST phylogroups or the hspEuropeN, hspEuropeS, hspMiscAmerica, and hspAfrica1NAmerica, as described recently by fineSTRUCTURE (Supplementary Table 1). The ANI-BLAST IA and II clades contained more genomes of *H. pylori* isolated in Mexico from gastric cancer and intestinal metaplasia than from the other gastric pathologies. These findings reflect the population structure of the Mexican citizens and show that *H. pylori* genomes are carriers of segments of European and African ancestries.

**Figure 4:**
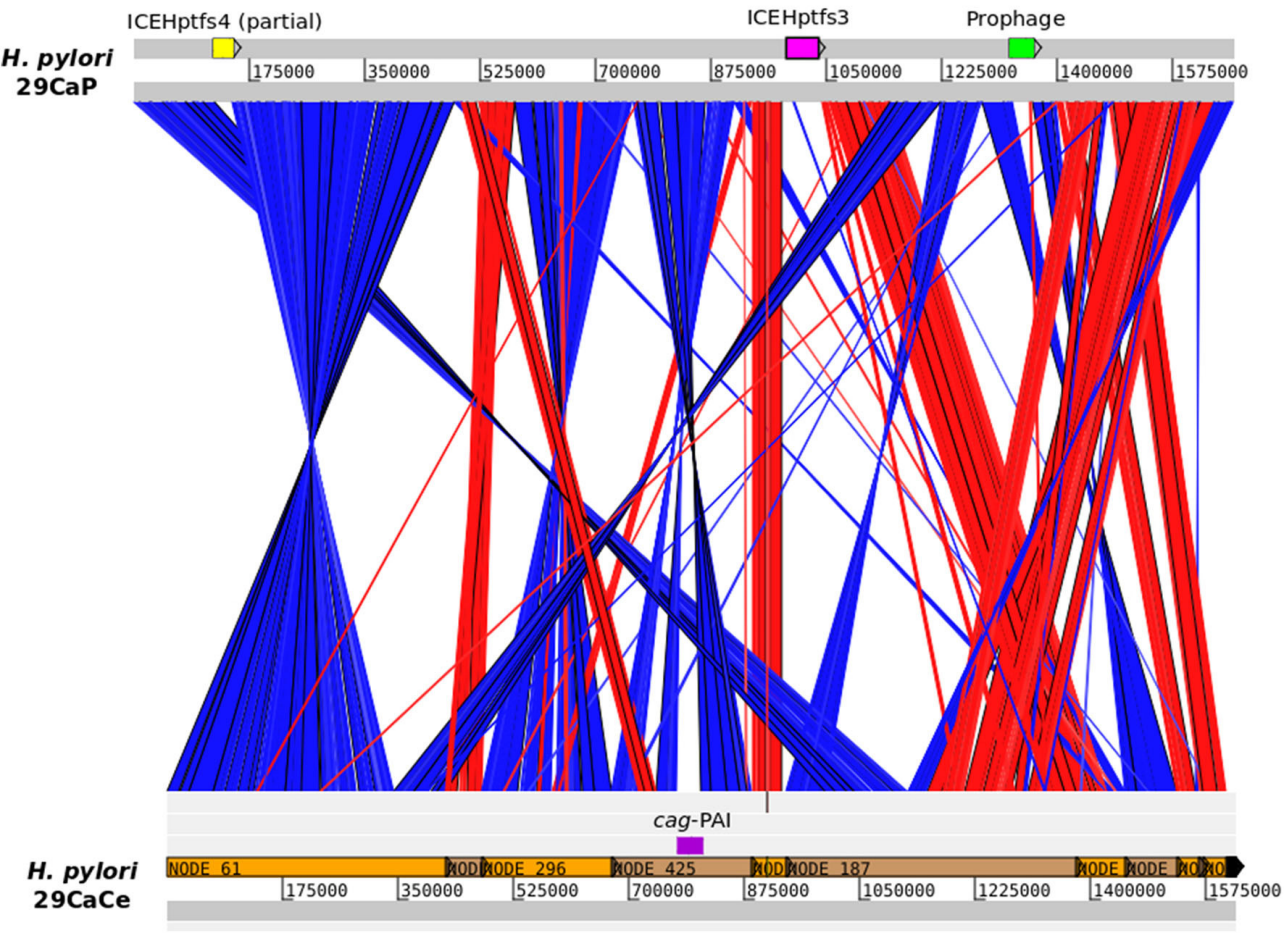
Comparison of *H. pylori* 29CaP and 29CaCe genomes. Linear maps of *H. pylori* genomes are connected by vectors matching the similar regions between them. Red vectors represent sequences with the same orientation, whereas blue vectors represent regions with inverted sequences. *cag*-PAI, ICEs, and prophages are signaled in the genomes. Images were generated using Artemis Comparison Tool v 18.1.0 (Sanger Institute) with blastn alignments.

We were able to analyze the genomes of *H. pylori* isolates from different gastric regions from three patients. For two of the patients, the population structure of their *H. pylori* isolates was identical. However, the genomes of *H. pylori* 29CaCe and 29CaP clustered in different ANI-BLAST groups. *H. pylori* 29CaCe was isolated from the center of the tumor in the same region as the recently sequenced 29CaP, isolated from the periphery of the tumor (Mucito-Varela et al., 2016). The *H. pylori* 29CaP genome clustered in ANI_BLAST I, whereas *H. pylori* 29CaCe genome did so in ANI_BLAST II. Furthermore, *H. pylori* 29CaCe harbors the *cag*-PAI and lacks ICEs, whereas *H. pylori* 29CaP lacks the *cag*-PAI and contains an ICE similar to ICEHptfs3. In addition, the *H. pylori* 29CaP genome encodes a prophage (Figure 4). These contrasting differences in the genome structure and phylogeographic origin of these strains suggest the presence of mixed infections with strains of different phylogeographic origins.

### Association of the Presence of ICEHptfs3 With Cytopathic Effects on AGS Cells

**Figure 5:**
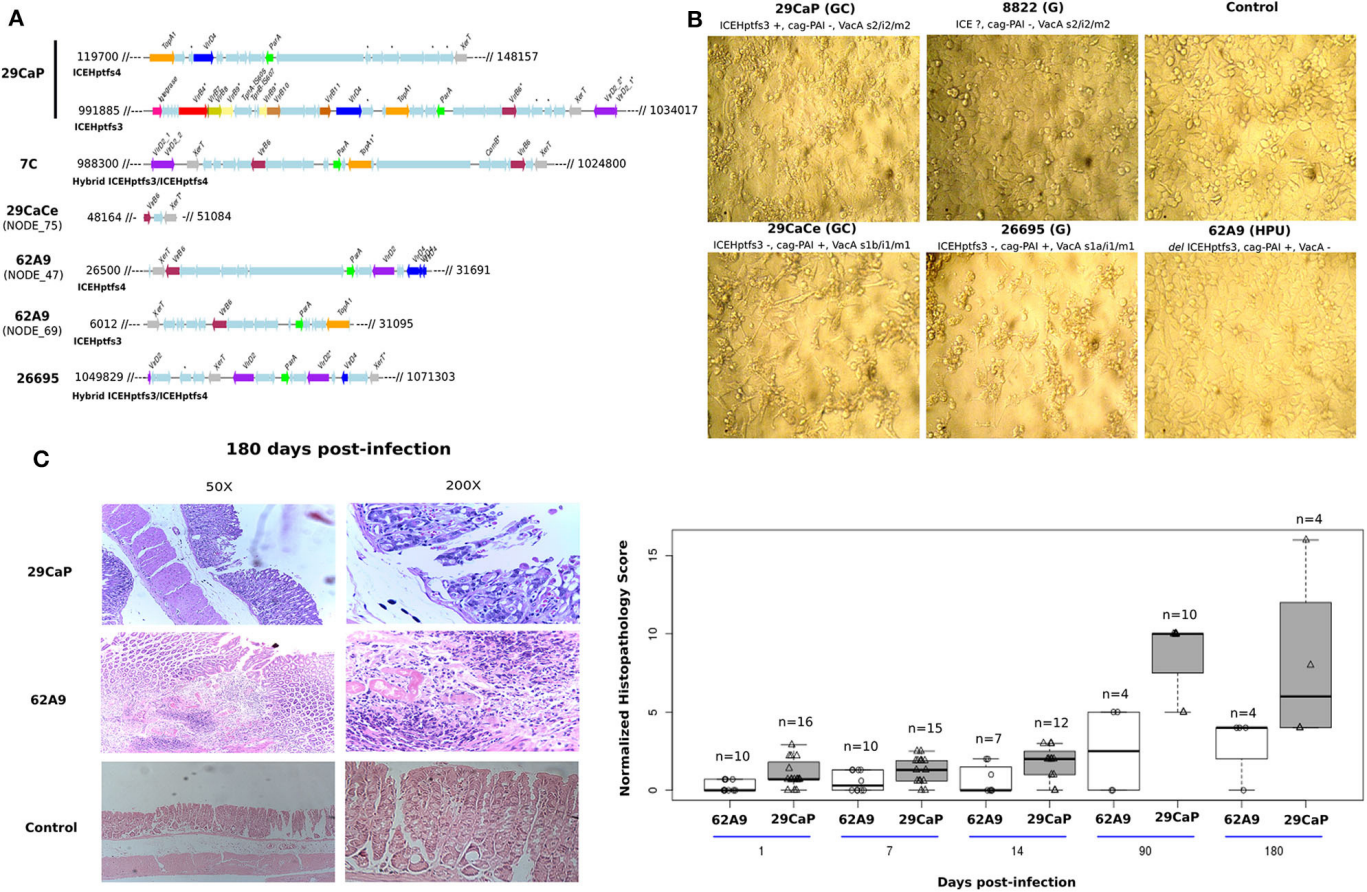
Evaluation of the functional effects of the complete ICEHptfs3. **(A)** *In silico* evaluation of TFSS completeness in *H. pylori* strains used for infection in AGS cells and mouse model. We show that *H. pylori* 29CaP has a complete TFSS located in the ICE element. **(B)** Cytopathic effects on AGS cells using strains with different *cag*-PAI and ICE genotypes**. (C)** Histopathological damage induced by *H. pylori* 29CaP and 62A9 in mice. Stomachs from infected mice were evaluated by hematoxylin and eosin staining. Scores were normalized, dividing the individual score of each infected mouse by the average score of their respective control group.

Selected *H. pylori* strains with different *cag*-PAI and ICE genotypes were put in contact with AGS cells (Figure 5A). This cell line was used because of its gastric epithelial origin, and despite being from adenocarcinoma, it still has the potential to study loss of cell adherence; therefore, it has been widely used to assess *H. pylori* cytopathic effects. Furthermore, we could minimize vacuolization effects due to VacA because this cell line has been observed to react poorly to VacA (Schneider et al., 2011). The multiplicity of infection used in our experiments was previously demonstrated to elicit morphologic and proteomic changes in AGS cells (Castillo-Rojas et al., 2009).

At 48 h, we observed a strong cytopathic effect in cells infected with *H. pylori* strain 29CaP, which only carries ICEHptfs3 but no *cag*-PAI, characterized by cellular disaggregation and vacuolization. In contrast, the strains carrying *cag*-PAI elicited the well-known hummingbird phenotype, whereas strains with no *cag*-PAI nor ICE did not produce any morphology change on the AGS cells (Figure 5B).

### Histopathological Evaluation of Infected Mice

From 36 and 25 mice infected with *H. pylori* 29CaP and 62A9 strains, respectively, we observed that infection with both strains produced an inflammatory pattern of chronic gastritis and epithelial erosion (Figure 5C). These effects were shown at higher rates at 90 and 180 days postinfection. The effect was measured by quantifying a histopathological score that was greater in the *H. pylori* 29CaP group than *H. pylori* 62A9. The damage was independent of bacterial persistence, as both strains were detected along the 180 days of infection (Table 4). Interestingly, *H. pylori* 29CaP had less ability to colonize the stomachs of the mice, contrasting with the higher histopathological score.

**Table 4:** Comparison of *H. pylori* infection outcomes in BALB/c.

**Days post infection**	**29CaP (*****cag*****-PAI -, ICEHptfs3, VacA s2/i2/m2)**			**62A9 (*****cag*****-PAI** **+**, ***del*** **ICEHptfs3, VacA -)**		
	**Infection rate IHC**	**Infection rate PCR-hybridization**	**Average histopathological score**	**Infection rate IHC**	**Infection rate PCR-hybridization**	**Average histopathological score**
1	0/8	2/8	1.0	1/6	2/6	0.3
7	1/8	4/8	1.3	1/6	4/6	0.6
14	2/7	3/8	1.8	3/6	4/6	0.7
90	1/6	0/8	9.6	1/6	4/6	2.5
180	2/7	3/8	8.0	2/6	4/6	3.0

## Discussion

In this work, we compared the current available *H. pylori* genomes isolated from different geographic areas and pathologies to identify differences between gene content and phylogeographic origin in the strains from gastric cancer and those from other pathologies. We extrapolated this relationship to a set of *H. pylori* genome isolates in Mexico from different pathologies.

The function of the proteins shared between gastric cancer strains suggests that, during the progression to pathogenesis, *H. pylori* actively remodels its genome and contends with oxidative stress to adapt to the host's response and persist in the stomach. We found that a gene annotated in some genomes as an integrase was present in more than 80% of the genomes of *H. pylori* isolated from gastric cancer; the second gene found at high frequency was a DNA topoisomerase I. Both are encoded within a genomic island of the *H. pylori* 29CaP genome named ICEHptfs3 (Fischer et al., 2014). Previous comparative genomic analyses using microarray technologies have identified restriction–modification systems and T4SS, as well as hypothetical proteins from ICEs, as potential biomarkers of disease. Within these genes, integrase and DNA topoisomerase I have been found as gastric cancer-specific genes (Romo-González et al., 2009; You et al., 2012). However, when we tried to associate the presence of the single genes with pathology, we did not find them useful, as integrase and DNA topoisomerase I did not show a significant correlation in the genomes of *H. pylori* isolates in Mexico but with *H. pylori* genomes from other regions of the world, which could be attributed to the great variability of ICE elements reported to date (Delahay et al., 2018). Similarly, we cannot rule out the possibility that some genes might be missing when working with draft sequences, due to mis-assembly or mis-annotation. Therefore, future studies that allow the characterization of the ICE sequences with better precision must be performed either by amplification or targeted sequencing. However, these considerations allowed us to generate the hypothesis that the presence of intact ICE segments in *H. pylori* genomes might account for virulence and gastric disease pathogenesis, rendering their presence as a better target to study biomarkers for gastric cancer risk.

We found that the *H. pylori* genomes isolated from gastric cancer in Mexico encode a potential complete ICE in higher proportion than the genomes of *H. pylori* isolated from other gastric pathologies. This suggests that the screening of ICE completeness might be a predictor of gastric cancer risk. We could not exclude the presence of ICEs in the genomes of *H. pylori* isolated from other gastric pathologies because the development of gastric cancer is preceded by a series of stages starting from gastritis, gastric atrophy, and intestinal metaplasia, or even by the gastric ulcer. The results we present here come from an observational cross-sectional study of *H. pylori* genomes isolated at a specific stage of gastric pathology. To our knowledge, there are currently few *H. pylori* genomes of strains isolated from the same patient during their evolution to gastric cancer. This is the case of the pair of genomes of *H. pylori* kx1 (HPKX_438_AG0C1) and *H. pylori* Kx2 (HPKX_438_CA4C1), which were also included in our validation set 3. Both genomes are highly fragmented draft sequences, and some POGs associated with gastric cancer were not annotated. However, when we recruited the contigs with similarity to ICEs in those genomes, we found that ICEHptfs4b has high length coverage in both genomes (Giannakis et al., 2008). These results show that ICEs might be present from the early stages of gastric pathology.

On the other hand, the dynamics of the mobile genetic elements must be considered when genotyping a strain, as seen in the genomes of 29CaP and 29CaCe. These two strains were isolated from the same patient at different tumor sites but are genetically different in gene content and genome population structure. The presence of multiple lineages of *H. pylori* within the same patient has been evaluated by studying the diversity of genotypes of VacA and CagA virulence factors. Those studies have found a diversity of these genotypes in colonies isolated from the same patients (Morales-Espinosa et al., 1999; López-Vidal et al., 2008).

In this study, the cytopathic effects and histopathological damage caused by *H. pylori* with different genetic compositions were evaluated. Because *cag*-PAI, *vacA*, and ICEs might be the major drivers of pathogenesis, strains with different genotypes of these elements were selected. The morphologic changes induced in AGS cells by *H. pylori* strains give insights into the molecular mechanisms involved in gastric carcinogenesis; for instance, the loss of adherence and cell elongation correlates with the invasion capability of the cancer hallmarks, whereas the vacuolization observed is related to increased cell death that may lead a persistent proliferation signal. In mouse infection, both strains used are carriers of the considered less virulent *vacA* genotypes or missing this gene; the damage was inferred to be driven by T4SS either by ICEs or *cag*-PAI. However, there is still the possibility that other genes of unknown function could contribute to cell damage.

*H. pylori* 29CaP, which harbors a complete ICEHptfs3 but lacks *cag*-PAI, elicited greater histopathological scores than *H. pylori* 62A9 strain, which contains a complete *cag*-PAI locus and an incomplete ICE element with no T4SS. Furthermore, evidence of tissue hyperplasia was observed in some mice infected with *H. pylori* 29CaP (data not shown). These results are consistent with the pathology associated with these strains, as *H. pylori* 29CaP was isolated from advanced gastric cancer lesions and *H. pylori* 62A9 from hemorrhagic peptic ulcer. In agreement with this evidence, it was previously reported that strains expressing CagA and VacA were found more prone to develop non- or delayed-healing ulcers than CagA and VacA negative strains, even when inflammation was caused by both types of strains (Konturek et al., 1999; Werawatganon, 2014). However, we can still partially link our results with the publications using mouse models because ICEHptfs3 status had not been assessed in those studies. Even the widely studied *H. pylori* strain SS1, which is CagA positive and lacks *cag*-PAI functionality, has been demonstrated to be a non-ICE carrier (Kersulyte et al., 2003) (data not shown). Despite its great variability, it has been stated that a mouse model might aid to study the effect of non-*cag*-PAI-related virulence factors because inflammatory effects are observed even in strains with non-functional or deleted *cag*-PAI (Konturek et al., 1999); therefore, our study is opening new insights into ICE functionality using mouse models, but studies using mutant and complementary strains of ICEs must be designed. This latter experiment represents a challenge because of the difficulty of genetically manipulated *H. pylori* genome due to the diversity of the restriction–modification systems.

The role of ICEs in gastric cancer pathogenesis could be associated to inflammation induction in the gastric epithelium (Waskito et al., 2018) because T4SS encoded by these elements promotes the induction of interleukin 8 independent from the presence of *cag*-PAI (Kersulyte et al., 2009), reinforced when the determinant is present (Silva et al., 2017). In that sense, we did not find any association between *cag*-PAI status and ICE presence, given that all the pathology groups can be both *cag*-PAI and ICE positive or negative, although we observed a tendency toward a high-frequency *cag*-PAI-positive/complete ICE-positive *H. pylori* genomes in strains isolated in Mexico and a high proportion of *cag*-PAI-positive/incomplete ICE-positive genomes in strains from gastritis. We suggest that the secretion–translocation of protein effectors is enhanced by the presence of both *cag*-PAI-T4SS and ICE-T4SS. This mechanism might be involved in gastric pathogenesis; for example, the secretion of the cell translocation kinase A was shown to be dependent on the T4SS of ICEHptfs3, promoting interleukin 8 secretions in gastric epithelial cells (Alandiyjany et al., 2017). Other protein effectors could be translocated by the T4SS of *H. pylori*, as predicted by *in silico* analyses of *H. pylori* genomes (Wang et al., 2014). Based on our results, we can hypothesize that *H. pylori* strains carrying a complete ICE are capable of inducing cell damage *in vivo* and *in vitro*, even in the absence of *cag*-PAI. Both elements might act independently, but a synergistic effect cannot be excluded.

Given that the secretion of virulence factors, including the expression of ICEs, might reflect a host–pathogen adaptation (Kodaman et al., 2014), it is necessary to study the ancestry of both the pathogen and the host. Because we were unable to obtain information on the hosts from databases, we assessed the relation between ICE presence and the phylogeographic origin of *H. pylori* strains. At that point, we faced the lack of annotated genes used in *H. pylori* typing by MLST, mainly because most of the genomes studied were draft sequences. Therefore, we decided to cluster *H. pylori* genomes by ANI, defining groups that correlated with the phylogeographic origin of the strains. The ANI represents the mean of identity values between the homologous regions shared by two genomes. Generally, the intraspecies ANI value varies between 80 and 100% in a distribution shifting toward values higher than 96%, whereas values below 95% are proposed to define species with identity correlation higher than 98% in the *16S rRNA* (Kim et al., 2014). ANI values corresponding to the genomes of *H. pylori* analyzed in this work ranged from 92 to 99%, with lower ANI values among the groups from Asian and African genomes. This suggests that there has been little genetic exchange between genomes of *H. pylori* strains in these regions. It also points to the presence of different conditions of association between the bacteria and their host. These observations agree with the reports in the literature. Some studies indicate that, by inferring the population structure of 29 *H. pylori* genomes, genomes of *H. pylori* belonging to hpEastAsia have lower numbers of chromosomal regions acquired from hpAfrica and hpEurope than those transmitted to the same groups. This might be due to less efficient DNA genetic mechanisms for importing or a different selective environment preventing DNA imports in the past (Yahara et al., 2013). We did not observe any association between the phylogeographic origin and the presence of an ICE type likely because ICEs are found as hybrid regions of the four ICE types described. However, in the genomes of *H. pylori* isolated from gastric cancer in Mexico, we observed an increased frequency of sequences grouped in the ANI-Blast II group related to an HpAfrica1 origin. When we analyzed the segments similar to the four ICE types, we observed that ICEHptfs3 was absent or highly fragmented in HpAfrica1 strains. These results are consistent with the report by Delahay et al. (2018), who described a modular acquisition of ICE elements and also reported a group of HpAfrica1 strains lacking the main regions of ICEHptfs3 but containing ICEHptfs4a-c. Future works must study the ancestry of both *H. pylori* and its host to better understand their effect on the expression of ICEs and other virulence factors.

In conclusion, *H. pylori* ICEs are targets to identify potentially helpful disease biomarkers of gastric cancer. Besides the genetic characterization of these elements, functional analyses are needed because many genes encoded in them do not have predicted functions by homology inference. The *in vitro* and *in vivo* effects observed in this work suggest that these elements are interesting candidates that need to be addressed in future studies by a “multiomic” approach. In addition, recombination events between ICEs might drive the gain or loss of genes that would moderate the host response, contributing in this way to pathogenesis. This latter phenomenon could be modulated by the compatibility of *H. pylori* genomic population structure during multiple infections.

## Data Availability Statement

The datasets generated for this study can be found in the Whole Genome Shotgun project has been deposited at DDBJ/ENA/GenBank (https://www.ncbi.nlm.nih.gov/bioproject) under the accession PRJNA513874.

## Ethics Statement

The animal study was reviewed and approved by Facultad de Medicina Veterinaria y Zootecnia, Universidad Nacional Autónoma de México.

## Author Contributions

EM-V and YL-V conceived and designed the experiments. EM-V performed the experiments. EM-V, GC-R, JC, and YL-V analyzed the data. EM-V, GC-R, JC, and YL-V wrote the manuscript. All authors reviewed and approved the manuscript.

## Conflict of Interest

The authors declare that the research was conducted in the absence of any commercial or financial relationships that could be construed as a potential conflict of interest.
