# Supplementary material for: Integrative and Conjugative Elements of Helicobacter pylori Are Hypothetical Virulence Factors Associated With Gastric Cancer
Source: Front Cell Infect Microbiol. 2020 Oct 19;10:525335. doi: 10.3389/fcimb.2020.525335 (PMC7604443; doi:10.3389/fcimb.2020.525335)
Supplement: Supplementary file 1 [file Data_Sheet_1.docx]

Integrative and Conjugative Elements of *Helicobacter pylori* Are Hypothetical Virulence Factors Associated With Gastric Cancer

Eduardo Mucito-Varela, Gonzalo Castillo-Rojas, Juan J. Calva and Yolanda López-Vidal

Supplementary Material

**a**


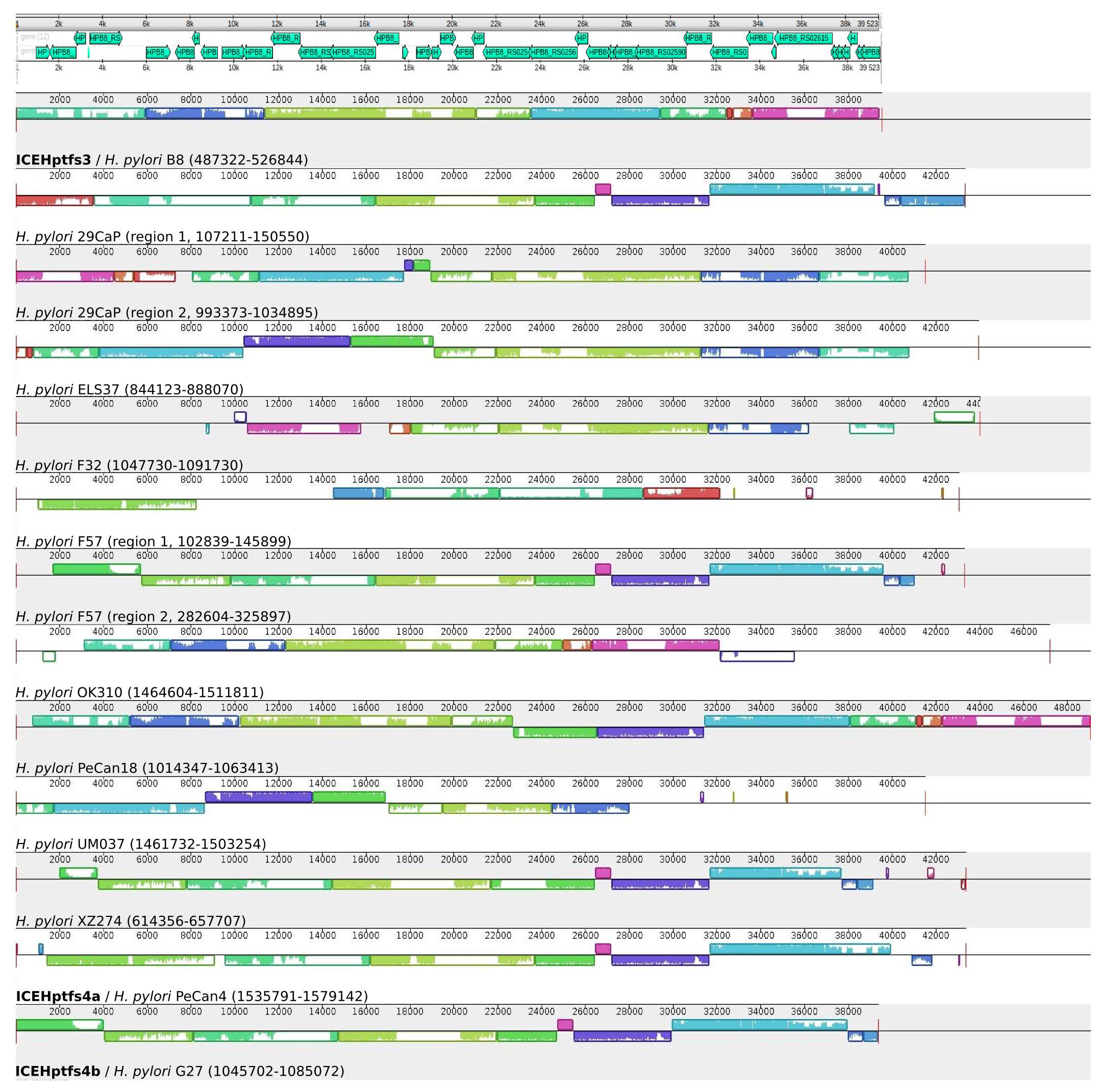


**b**


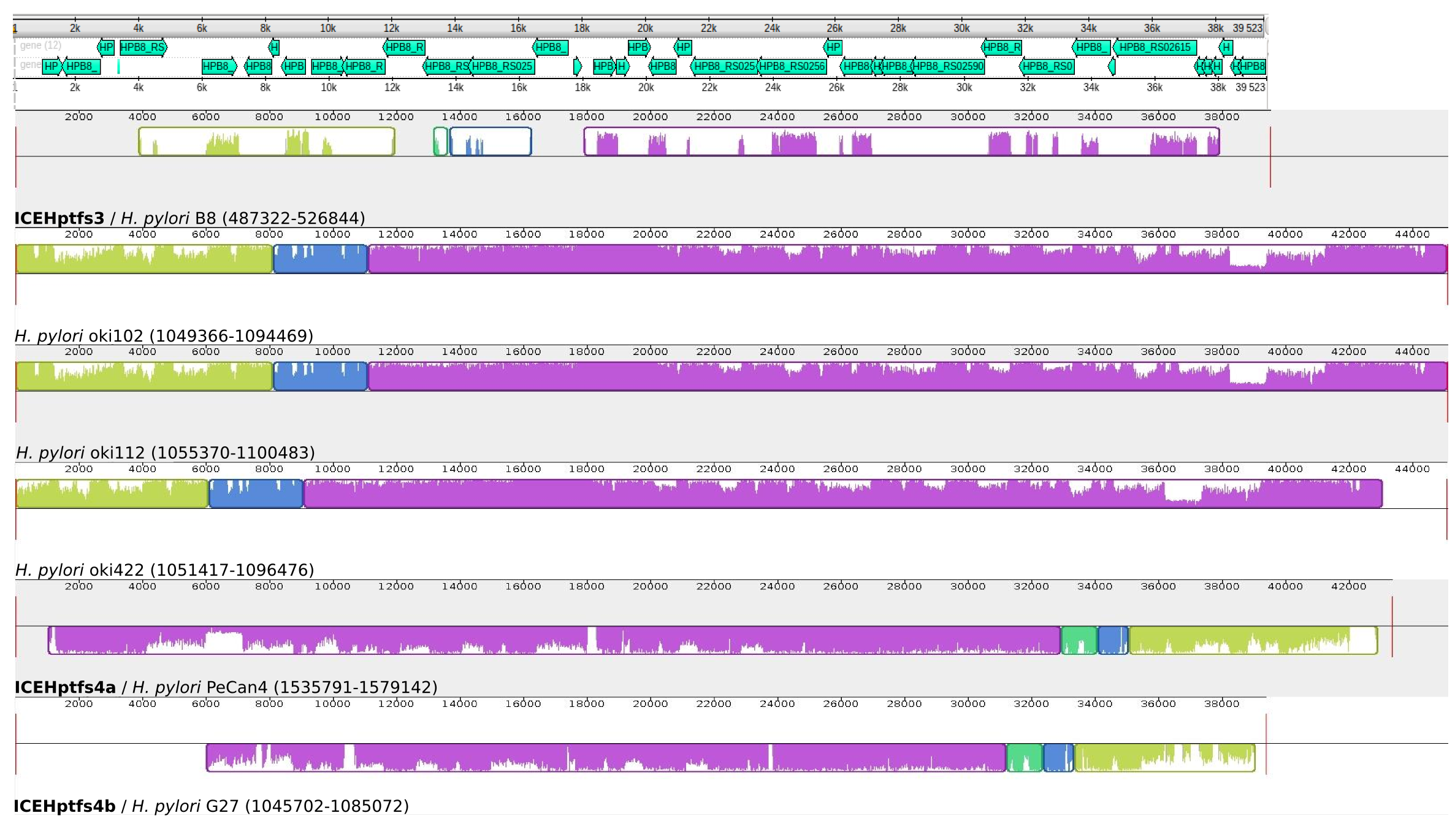


**c**


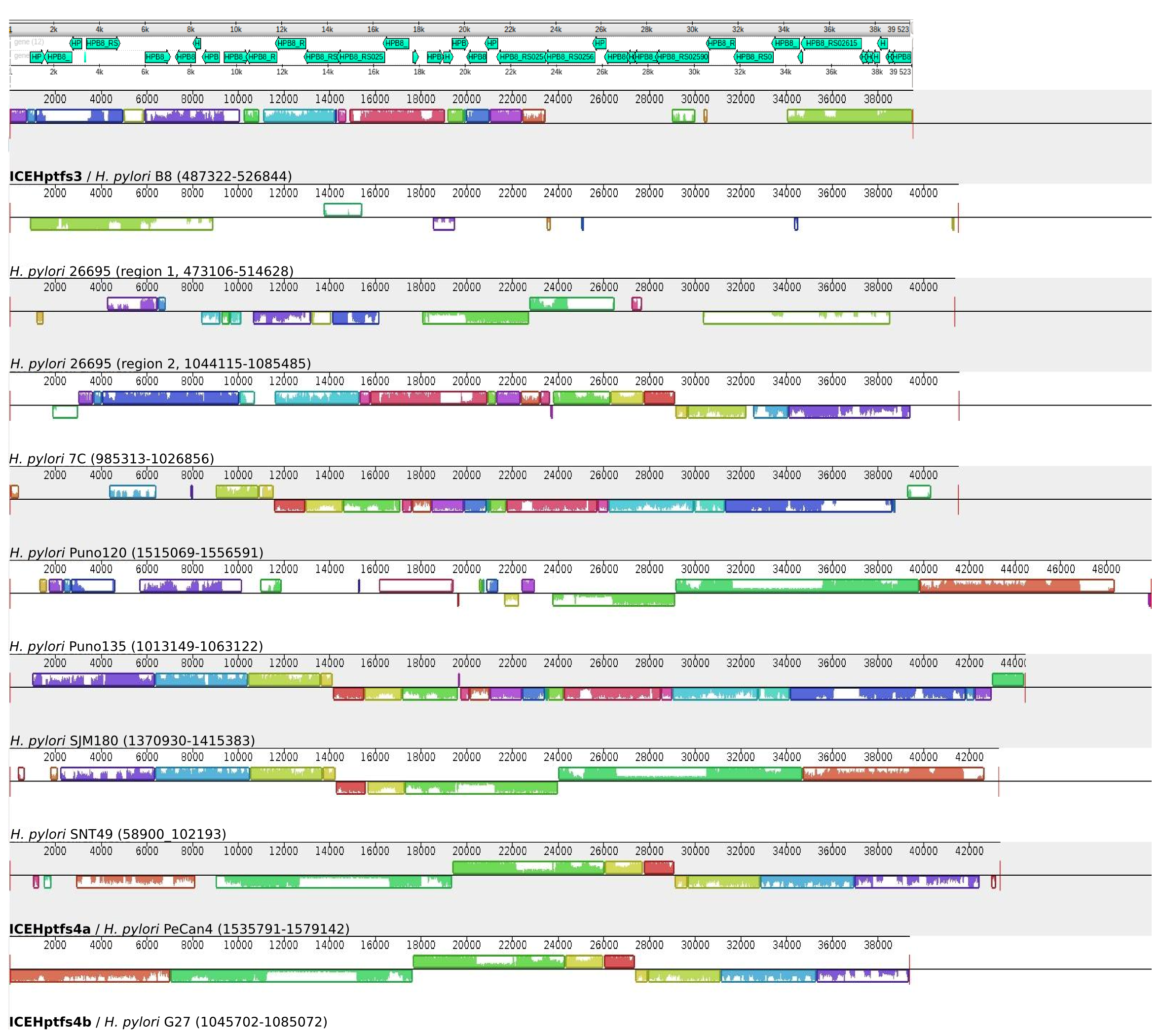


**d**


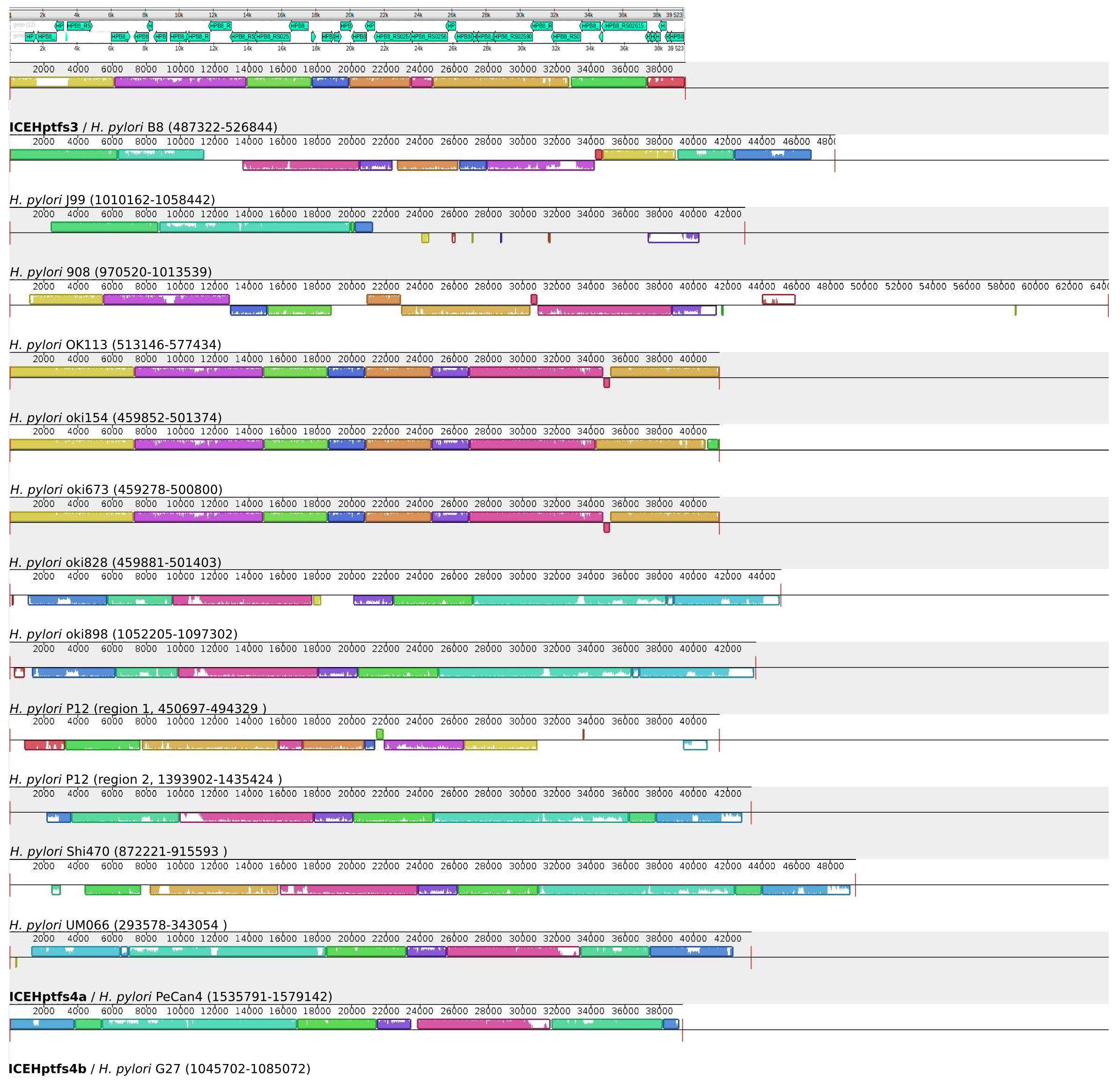


**e**


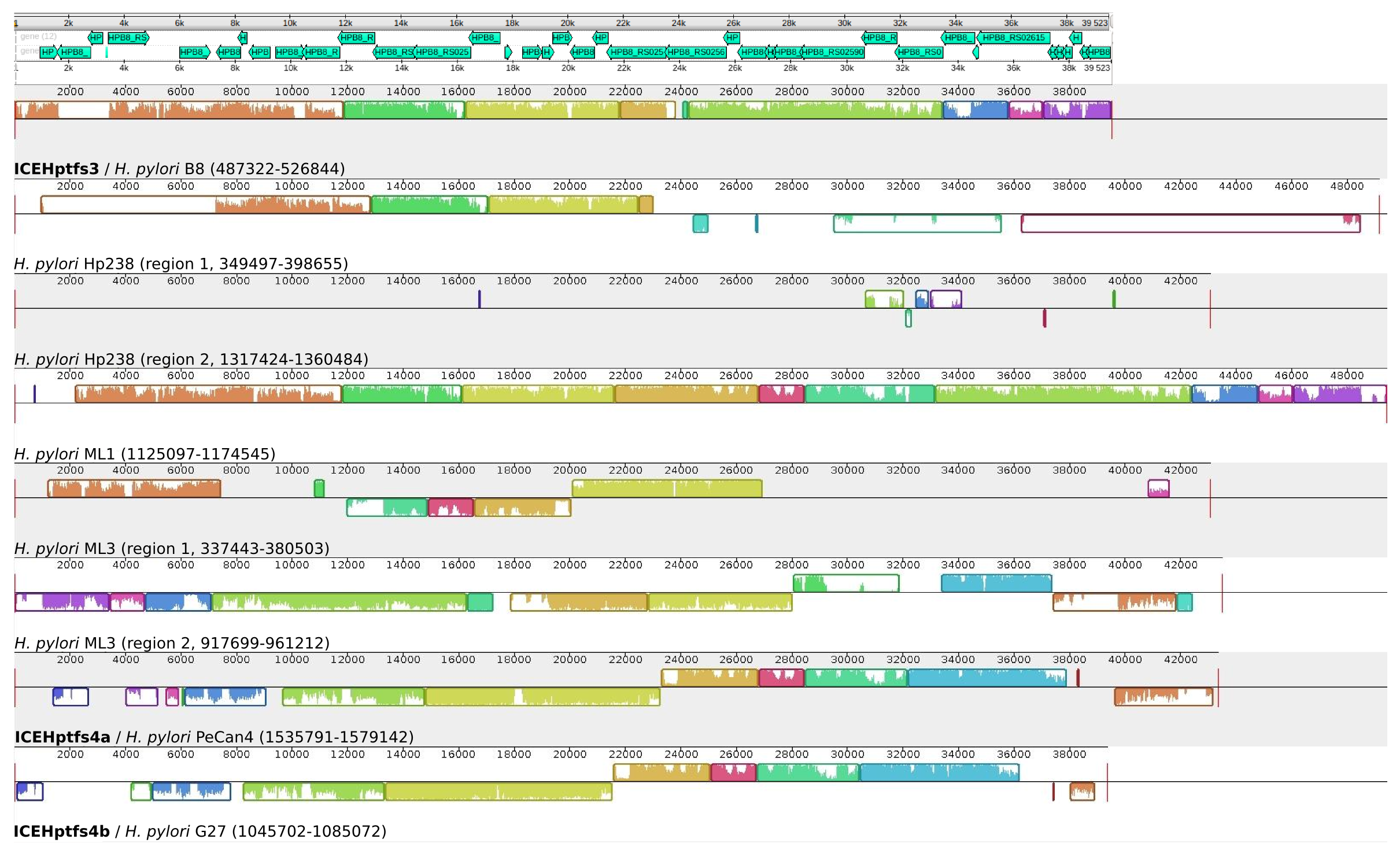


**Supplementary Figure 1.** **Integrative and Conjugative Elements in complete genomes of *H. pylori***. Multiple sequence alignments show regions similar between *H. pylori* complete genomes and the reference ICE sequences taken from Fischer et al., 2014. Alignments were performed with the progressive Mauve algorithm from Mauve version 2.4.0 with the default parameters. Each row show the region similar to ICEs in the different *H. pylori* genomes. The boxes in color represents the regions similar at least to another sequence while the color lines inside the boxes represent the degree of similarity between regions. The complete genomes that does not appear in the figure had no positive alignment. At the top of the figure, a representation of the genes encoded in the ICEHptfs3 element obtained using UGENE genome analysis suite version 1.3. The images were assembled and edited in Inkscape version 0.92.1, to annotate the name of the strains and the position in the genome of the region showed. **(a)** Genomes of *H. pylori* isolated from gastric cancer. **(b)** Genomes of *H. pylori* isolated from chronic atrophic gastritis. **(c)** Genomes of *H. pylori* isolated from peptic ulcer. **(d)** Genomes of *H. pylori* isolated from gastritis. **(e)** Genomes of *H. pylori* isolated from MALT lymphoma.

**
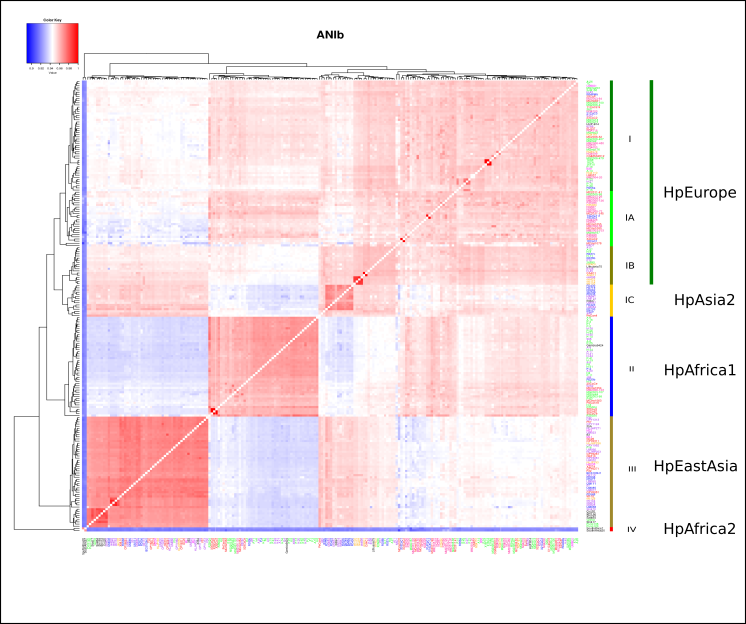
Supplementary Figure 2. Clustering of the *H. pylori* genomes by Average Nucleotide Identity Using BLAST.** The figure was obtained as described in materials and methods. Square colors represent the average percent identity between all pairs of segments matched in each pair of genomes, the scale shown at the left top of the figure indicates blue a percent identity of 90% and red a percent identity of 100%. The names of the *H. pylori* strains indicate the pathology associated as follows: red = gastric cancer, deep pink = precancerous lesions, orange = chronic atrophic gastritis, green = gastritis, purple = peptic ulcer, magenta = MALT lymphoma, blue = dyspepsia or asymptomatic. At the right of each cluster the correlation with the MLST population structure reported in the literature.

| **Supplementary table 1.** Genomes used in ANI-BLAST analysis and validation set 3 | | | | |
| --- | --- | --- | --- | --- |
| **Strain** | **Pathology** | **ANI_BLAST** | **MLST**** | **fineSTRUCTURE***** |
| 7C * | G | I | hpEurope |  |
| 29CaP * | GC | I | hpEurope |  |
| A-14* | G | I | hpEurope | hspEuropeN |
| A-26* | G | I | hpEurope | hspEuropeN |
| B38* | L | I | hpEurope | hspEuropeN |
| CGIMSS-2012* | GC | I | hpEurope |  |
| ELS37* | GC | I | hpEurope | hspEuropeS |
| H-27* | PU-G | I | hpEurope | hspEuropeN |
| H-43* | PU-DU | I | hpEurope | hspEuropeN |
| 62A9 | PU | I |  |  |
| 53C1 | PU | I |  |  |
| 11CaC2-9 | GC | I |  |  |
| 23CaC2 | GC | I |  |  |
| 21CaC3 | D | I |  |  |
| 16F10 | G | I |  |  |
| 16A8 | G | I |  |  |
| 16C4 | G | I |  |  |
| 36C | G | I |  |  |
| HUP-B14 | NA | I | hpEurope | hspEuropeS |
| MC2006-52 | GC | I |  | hspEuropeS |
| MCms931 | GC | I |  | hspEuropeS |
| MG2005-100 | G | I |  | hspEuropeS |
| MG2006-4 | G | I |  | hspEuropeS |
| MG2006-407 | G | I |  | hspEuropeS |
| MG2006-479 | G | I |  | hspEuropeS |
| MGms2 | G | I |  | hspEuropeS |
| MGms13 | G | I |  | hspEuropeS |
| MGms15 | G | I |  | hspEuropeS |
| MGms23 | G | I |  | hspEuropeS |
| MGms176 | G | I |  | hspEuropeS |
| MGms203 | G | I |  | hspEuropeS |
| MM2004-20 | M | I |  | hspEuropeS |
| MM2006-480 | M | I |  | hspEuropeS |
| MU2003-84 | M | I |  | hspEuropeS |
| NQ315* | PC | I |  | hspEuropeColombia |
| NQ4053* | PC | I | hpEurope | hspEuropeColombia |
| NQ4076* | CAG | I | hpEurope | hspEuropeColombia |
| NQ4099* | Pc | I | hpEurope | hspEuropeColombia |
| NQ4110* | CAG | I |  | hspEuropeS |
| NQ4161* | CAG | I | hpEurope | hspEuropeColombia |
| NQ4200* | PC | I |  | hspEuropeColombia |
| NQ4216* | PC | I | hpEurope | hspEuropeColombia |
| P-15* | G | I |  | hspEuropeN |
| P-16* | G | I |  | hspEuropeN |
| P-23* | G | I |  | hspEuropeN |
| P-30* | G | I |  | hspEuropeN |
| P-74* | G | I |  | hspEuropeN |
| R046Wa* | A | I |  | hspEuropeN |
| R056a* | A | I |  |  |
| SJM180* | G | I |  | hspEuropeS |
| UM037* | GC | I |  |  |
| UMBG1* | L | I |  |  |
| 18CaC1 | D | IA |  |  |
| 34CaC2 | GC | IA |  |  |
| 14CaC1 | D | IA |  |  |
| 14CaC1-2 | D | IA |  |  |
| MC2011-41 | G | IA |  | hspEuropeS |
| MC2011-145 | GC | IA |  | hspMiscAmerica |
| MCms1055 | GC | IA |  | hspEuropeS |
| MCms1078 | GC | IA |  | hspEuropeS |
| MCms1080 | GC | IA |  | hspEuropeS |
| MG2005-98 | G | IA |  | hspEuropeS |
| MGms167 | G | IA |  | hspMiscAmerica |
| MM2003-103 | M | IA |  | hspEuropeS |
| MM2005-72 | M | IA |  | hspMiscAmerica |
| MM2005-126 | M | IA |  | hspEuropeS |
| MM2006-56 | M | IA |  | hspEuropeS |
| MM2012-26 | M | IA |  | hspEuropeS |
| MU2004-2 | M | IA |  | hspEuropeS |
| NAD1* | PU-DU | IA | hpEurope |  |
| NQ352* | PC | IA |  |  |
| NQ367* | PC | IA |  |  |
| NQ392* | PC | IA |  | hspEuropeColombia |
| NQ4228* | PC | IA | hpEurope | hspEuropeColombia |
| PZ5026* | PC | IA |  | hspEuropeColombia |
| PZ5056* | PC | IA |  | hspEuropeColombia |
| PZ5080* | CAG | IA |  | hspEuropeColombia |
| PZ5086* | PC | IA |  | hspEuropeColombia |
| 26695* | G | IB | hpEurope | hspEuropeN |
| A-11* | PU-DU | IB |  | hspEuropeN |
| A-27* | G | IB | hpEurope | hspEuropeN |
| AG0C1* | CAG | IB |  | hspEuropeN |
| B128* | PU-G | IB | hpEurope |  |
| CA4C1* | GC | IB |  |  |
| H-9* | G | IB | hpEurope | hspEuropeN |
| H-11* | G | IB | hpEurope | hspEuropeN |
| H-45* | PU-DU | IB | hpEurope | hspEuropeN |
| HPAG1* | CAG | IB | hpEurope | hspEuropeN |
| Lithuania75 | NA | IB | hpEurope | hspEuropeN |
| oki102* | CAG | IB |  |  |
| oki112* | CAG | IB |  |  |
| oki422* | CAG | IB |  |  |
| oki898* | PU-DU | IB |  |  |
| P12* | PU-DU | IB | hpEurope | hspEuropeN |
| R037c* | A | IB |  |  |
| R038b* | A | IB |  | hspEuropeN |
| A45* | PU-G | IC |  |  |
| FD423* | D | IC | hpAsia2 | hpAsia2 |
| FD430* | D | IC |  | hpAsia2 |
| FD535* | D | IC | hpAsia2 | hpAsia2 |
| FD662* | D | IC |  |  |
| FD703* | D | IC | hpAsia2 | hpAsia2 |
| FD719* | D | IC | hpAsia2 |  |
| India7 | NA | IC | hpAsia2 | hpAsia2 |
| NAB47* | PU-DU | IC | hpAsia2 | hpAsia2 |
| PeCan4* | GC | IC |  | Hybrid |
| R32b* | A | IC |  | hspEuropeN |
| SNT49 | A | IC |  | hpAsia2 |
| UM067* | PU | IC |  | hpAsia2 |
| UM084* | PU | IC |  | hpAsia2 |
| UM114* | PU | IC |  | hpAsia2 |
| 908* | PU-DU | II | hpAfrica1 |  |
| A-4* | PU-DU | II | hpAfrica1 |  |
| A-5* | PU-G | II | hpAfrica1 | hspAfrica1NAmerica |
| A-6* | G | II | hpAfrica1 | hspAfrica1NAmerica |
| A-8* | G | II | hpAfrica1 | hspAfrica1NAmerica |
| A-16* | G | II | hpAfrica1 | hspAfrica1NAmerica |
| A-17* | PU-G | II | hpAfrica1 | hspAfrica1NAmerica |
| A-20* | PU-DU | II | hpAfrica1 | hspAfrica1NAmerica |
| Gambia9424 | NA | II | hpAfrica1 | hspAfrica1WAfrica |
| H-4* | G | II | hpAfrica1 | hspAfrica1NAmerica |
| H-3* | G | II | hpEurope | hspAfrica1NAmerica |
| H-6* | G | II | hpEurope |  |
| H-16* | PU | II |  |  |
| H-19* | G | II | hpAfrica1 | hspAfrica1NAmerica |
| H-21* | G | II | hpAfrica1 | hspAfrica1NAmerica |
| H-23* | G | II | hpAfrica1 | hspAfrica1NAmerica |
| H-24* | PU-G | II | hpAfrica1 | hspAfrica1NAmerica |
| H-30* | PU-G | II | hpAfrica1 | hspAfrica1NAmerica |
| H-34* | G | II | hpAfrica1 | hspAfrica1NAmerica |
| H-41* | PU-DU | II | hpEurope | hspAfrica1NAmerica |
| 30CaCe | GC | II |  |  |
| 52A2 | PU | II |  |  |
| 30CaF2 | GC | II |  |  |
| 30Ca+5 | GC | II |  |  |
| 29CaCe | GC | II |  |  |
| J99* | PU-DU | II | hpAfrica1 | hspAfrica1NAmerica |
| MCms1054 | GC | II |  | hspMiscAmerica |
| MCms1063 | GC | II |  | hspMiscAmerica |
| MG2003-98 | G | II |  | hspMiscAmerica |
| MG2003-107 | G | II |  | hspMiscAmerica |
| MGms44 | G | II |  | hspMiscAmerica |
| MM2006-103 | M | II |  | hspMiscAmerica |
| P-1* | G | II |  |  |
| P8* | G | II |  | hspAfrica1NAmerica |
| P-2* | G | II |  | hspAfrica1NAmerica |
| P-3* | G | II |  | hspAfrica1NAmerica |
| P-4* | D | II |  |  |
| P-11* | G | II |  | hspAfrica1NAmerica |
| P-13* | G | II |  | hspAfrica1NAmerica |
| P-25* | G | II |  |  |
| P-26* | G | II |  | hspAfrica1NAmerica |
| P-41* | G | II |  |  |
| P-62* | G | II |  |  |
| PeCan18 | GC | II |  |  |
| PZ5004* | G | II |  | hspAfrica1NAmerica |
| PZ5024* | G | II |  |  |
| R030b* | A | II |  |  |
| 35A | NA | IIIA |  | hpEastAsia |
| 51* | PU-DU | IIIA | hpEAsia | hpEastAsia |
| 83 | NA | IIIA |  | hpEastAsia |
| 98-10* | GC | IIIA |  | hpEastAsia |
| BCS100H1* | A | IIIA |  |  |
| CPY1124* | PU-G | IIIA | hpEAsia | hpEastAsia |
| CPY1313* | PU-DU | IIIA | hpEAsia | hpEastAsia |
| CPY1962* | PU-G | IIIA |  | hpEastAsia |
| CPY3281* | PU-DU | IIIA |  | hpEastAsia |
| CPY6081* | GC | IIIA | hpEAsia | hpEastAsia |
| CPY6261* | GC | IIIA | hpEAsia | hpEastAsia |
| CPY6271* | GC | IIIA |  | hpEastAsia |
| CPY6311* | GC | IIIA | hpEAsia | hpEastAsia |
| F16* | G | IIIA | hpEAsia | hpEastAsia |
| F30* | PU-DU | IIIA | hpEAsia | hpEastAsia |
| F32* | GC | IIIA | hpEAsia | hpEastAsia |
| F57* | GC | IIIA | hpEAsia | hpEastAsia |
| FD506* | D | IIIA |  | hpEastAsia |
| FD568* | D | IIIA |  | hpEastAsia |
| FD577* | D | IIIA |  | hpEastAsia |
| GC26* | GC | IIIA |  |  |
| HLJHP253* | OU-G | IIIA |  | hpEastAsia |
| HLJHP256* | CAG | IIIA |  | hpEastAsia |
| HLJHP271* | PU-G | IIIA |  | hpEastAsia |
| HLJHP193* | CAG | IIIA |  | hpEastAsia |
| Hp238 | L | IIIA |  |  |
| ML1 | L | IIIA |  |  |
| ML3 | L | IIIA |  |  |
| OK113* | PU-DU | IIIA | hpEAsia | hpEastAsia |
| OK310* | GC | IIIA | hpEAsia | hpEastAsia |
| UM023* | PU | IIIA |  | hpEastAsia |
| UM032* | PU | IIIA |  | hpEastAsia |
| UM065* | PU | IIIA |  | hpEastAsia |
| UM066* | PU | IIIA |  | hpEastAsia |
| UM077* | PU | IIIA |  | hpEastAsia |
| UM085* | D | IIIA |  | hpEastAsia |
| UM111* | D | IIIA |  | hpEastAsia |
| XZ274* | GC | IIIA | hpEAsia | hpEastAsia |
| oki128* | CAG | IIIB |  |  |
| oki154* | PU-DU | IIIB |  |  |
| oki673* | PU-G | IIIB |  |  |
| oki828* | PU-DU | IIIB |  |  |
| UM038* | D | IIIB |  | hpEastAsia |
| Cuz20 | NA | IIIC | hspAmerind | hspAmerind |
| Puno120 | G | IIIC |  | hspAmerind |
| Puno135 | G | IIIC |  | hspAmerind |
| Sat464 | NA | IIIC |  | hspAmerind |
| Shi112 | NA | IIIC |  | hspAmerind |
| Shi169 | NA | IIIC |  | hspAmerind |
| Shi417 | NA | IIIC |  | hspAmerind |
| Shi470 | PU-G | IIIC |  | hspAmerind |
| v225d* | G | IIIC |  | hspAmerind |
| SouthAfrica7 | NA | IV |  | hpAfrica2 |
| SouthAfrica20 | NA | IV |  | hpAfrica2 |
| G = Gastritis, GC = Gastric cancer, L = MALT lymphoma, PU = Peptic Ulcer, PU-G = Gastric Ulcer, PU-DU = Duodenal ulcer, M = Intestinal Metaplasia, CAG = Chronic atrophic gastritis, A = Asimptomatic, D = Dyspepsia, PC = Precancerous lesion, NA = Not information available  ** Genome sequence included in Validation set 3*  ** *Data obtained from varios sources*  ****Data obtained from Thorell K, Yahara K, Berthenet E, Lawson DJ, Mikhail J, et al. (2017) Correction: Rapid evolution of distinct Helicobacter pylori subpopulations in the Americas. PLOS Genetics 13(4): e1006730.* | | | | |

| **Supplementary Table 2.** ICE and gastric cancer related POGs presence in *H. pylori* draft genomes from strains isolated in Mexico. | | | | | | | |
| --- | --- | --- | --- | --- | --- | --- | --- |
| **Strain** | **Pathology** | ***cag-*PAI** | **Complete ICE potential** | **ICE type*** | **Integrase** | **TopA 1** | **Reference** |
| 23CaC2 | GC | + | Y | Hybrid T3/T4 | - | + | This study |
| 29CaCe | GC | + | N | - | - |  | This study |
| 30CaCe | GC | + | Y | Hybrid T3/T4 | - | + | This study |
| 30CaF2 | GC | + | Y | Hybrid T3/T4 | - | + | This study |
| 30Ca+5 | GC | + | Y | Hybrid T3/T4 | - | + | This study |
| 34CaC2 | GC | + | N | - | + |  | This study |
| 11CaC2-9 | GC | + | N | - | + | + | This study |
| CG-IMSS-2012 | GC | + | N | - | + | - | Mendez-Tenorio, et al., 2014 |
| MC2006-52 | GC | + | Y | ICEHptfs3 | - | - | Thorell K, et al., 2017 |
| MC2011-145 | GC | + | Y | Hybrid T3/T4 | + | + | Thorell K, et al., 2017 |
| MCms1054 | GC | + | N | - | - |  | Thorell K, et al., 2017 |
| MCms1055 | GC | + | N | - |  | + | Thorell K, et al., 2017 |
| MCms1063 | GC | + | Y | Hybrid T4 | - | + | Thorell K, et al., 2017 |
| MCms1078 | GC | + | Y | Hybrid T3/T4 | - | + | Thorell K, et al., 2017 |
| MCms1080 | GC | + | N | - | - |  | Thorell K, et al., 2017 |
| MCms931 | GC | + | Y | Hybrid T4 | - |  | Thorell K, et al., 2017 |
| MM2004-20 | M | + | Y | Hybrid T3/T4 | - | + | Thorell K, et al., 2017 |
| MM2005-72 | M | + | Y | Hybrid T3/T4 | - | + | Thorell K, et al., 2017 |
| MM2006-103 | M | + | Y | Hybrid T3/T4 | - | + | Thorell K, et al., 2017 |
| MM2005-126 | M | + | N | - | - | - | Thorell K, et al., 2017 |
| MM2006-480 | M | + | Y | ICEHptfs3 | + | + | Thorell K, et al., 2017 |
| MM2006-56 | M | + | N | - | + | + | Thorell K, et al., 2017 |
| MM2012-26 | M | + | Y | Hybrid T4 | - | + | Thorell K, et al., 2017 |
| MU2003-84 | M | + | N | - | - | - | Thorell K, et al., 2017 |
| MU2004-2 | M | + | N | - | - | - | Thorell K, et al., 2017 |
| MM2003-103 | M | + | N | - | - | - | Thorell K, et al., 2017 |
| 36C | ChG | +/i | Y | Hybrid T3/T4 | - | + | This study |
| 16F10 | G | + | N | - | + | + | This study |
| 16A8 | G | + | N | - | + | + | This study |
| 16C4 | G | + | N | - | + | + | This study |
| MG2003-107 | G | + | N | - | - | - | Thorell K, et al., 2017 |
| MG2003-98 | G | + | Y | Hybrid T3/T4 | + | + | Thorell K, et al., 2017 |
| MG2005-100 | G | + | N | - | - | - | Thorell K, et al., 2017 |
| MG2005-98 | G | + | N | - | - | - | Thorell K, et al., 2017 |
| MG2006-4 | G | - | N | - | - | - | Thorell K, et al., 2017 |
| MG2006-407 | G | + | Y | Hybrid T3/T4 | + | - | Thorell K, et al., 2017 |
| MG2006-479 | G | + | N | - | + | + | Thorell K, et al., 2017 |
| MG2011-41 | G | + | N | - | + | + | Thorell K, et al., 2017 |
| MGms13 | G | + | N | - | - | + | Thorell K, et al., 2017 |
| MGms15 | G | + | N | - | + | - | Thorell K, et al., 2017 |
| MGms167 | G | + | Y | Hybrid T3/T4 | + | + | Thorell K, et al., 2017 |
| MGms176 | G | + | N | - | + | + | Thorell K, et al., 2017 |
| MGms2 | G | + | N | - | + | + | Thorell K, et al., 2017 |
| MGms203 | G | + | Y | Hybrid T3/T4 | - | + | Thorell K, et al., 2017 |
| MGms23 | G | + | N | - | - | + | Thorell K, et al., 2017 |
| MGms44 | G | + | N | - | - | + | Thorell K, et al., 2017 |
| 18CaC1 | D | + | N | - | + | - | This study |
| 14CaC1 | D | + | N | - | - | - | This study |
| 14CaC1-2 | D | + | N | - | - | - | This study |
| 21CaC3 | D | + | N | - | - | + | This study |
| 62A9 | PU | + | Y | Hybrid T3/T4 | - | + | This study |
| 53C1 | PU | + | Y | Hybrid T3/T4 | - | + | This study |
| 52A2 | PU | +/i | N | - | - | + | This study |
| GC = Gastric cancer, ChG = Chronic gastritis, M = Intestinal Metaplasia, G = Gastritis, D = Dyspepsia, PU = Peptic ulcer.  *cag*-PAI = *cag* pathogenicity Island, ICE = Integrative and conjugative element, TopA1 = DNA topoisomerase I.  + = presence  +/i = incomplete  - = absence  * Denotes the type of ICE element according to Fischer et al. (2014). | | | | | | | |

| **Supplementary Table 3.** BLAST Koala summary. | | | |
| --- | --- | --- | --- |
|  | **Genome data set** | | |
|  | **Exploratory** | **Validation 1** | **Validation 2** |
| Annotated proteins | 10/36 (27.8 %) | 2/25 (8%) | 8/25 (32%) |
| Functional categories | Nucleotide metabolism (4)**  Genetic Information Processing (1)  Environmental Information Processing  Cellular Processes (2)  Unclassified (3) | Genetic Information Processing (1)  Glycan biosynthesis and metabolism (1) | Genetic Information Processing (5)  Cellular Processes (2)  Environmental Information Processing (2)  Glycan biosynthesis and metabolism (1) |
| ** The number in parenthesis indicates the number of genes classified in the functional categories enlisted. | | | |
